# A scoping review of statistical methods used to report EORTC QLQ-C30 quality of life scores measured longitudinally

**DOI:** 10.1186/s12874-025-02622-1

**Published:** 2025-08-02

**Authors:** Rosie A. Harris, Chris A. Rogers, Jessica Harris, Eric Lim

**Affiliations:** 1 Bristol Trials Centre, Bristol Medical School, University of Bristol, Bristol, UK; 2 Royal Brompton and Harefield Hospitals, Guy’s and St Thomas’ NHS Foundation Trust, London, UK

**Keywords:** Statistical methodology, Statistical analysis, EORTC QLQ-C30, Quality of life, Longitudinal, Review

## Abstract

**Background:**

The European Organisation for Research and Treatment of Cancer Quality of Life Questionnaire Core 30 (QLQ-C30) is often used in cancer studies to assess patient reported quality of life over time. A number of challenges are faced when analysing scores derived from this questionnaire, and a multitude of statistical methods can be used. Whilst methods exist to overcome issues such as non-independence of repeated measurements and informative dropout, it is unclear how often these are implemented in practice. The aim of this scoping review was to comprehensively describe the statistical methods used to analyse longitudinal quality of life scores derived from the QLQ-C30 questionnaire.

**Methods:**

Two databases, MEDLINE and Embase, were searched for randomised controlled trials and prospective observational studies presenting statistical analyses of QLQ-C30 scores collected over time. Only studies published in English between 1 January 2021 and 31 December 2022 were included. Studies not reporting the statistical methods used were excluded. A REDCap database was developed to store extracted information and results are presented as descriptive summaries.

**Results:**

Two-hundred and seventy-one eligible studies were identified including 161 parallel group randomised controlled trials and 84 cohort studies. A linear mixed effects model was the most utilized analysis method, applied in 121/271 (45%) studies, followed by time to event analyses (54/271, 20%) and t-tests (44/271, 16%). Nearly one third of studies did not apply any longitudinal analysis method (82/271, 30%). Missing data due to death was accounted for in 23/271 (8%) studies. Statistical approaches did not differ greatly depending on the design of the study.

**Conclusions:**

There is no current consensus on the statistical method to analyse repeated QLQ-C30 scores. Many studies continue to ignore the longitudinal structure of repeated measures data, which could impact the interpretation of the data and conclusions drawn.

**Supplementary Information:**

The online version contains supplementary material available at 10.1186/s12874-025-02622-1.

## Background

Studies evaluating interventions for the treatment of cancer often assess health related quality of life (QoL) using the patient reported European Organisation for Research and Treatment of Cancer (EORTC) Quality of Life Questionnaires Core 30 (QLQ-C30) amongst others [1]. This questionnaire produces multiple scores assessing the different aspects of QoL. The QLQ-C30 measures global health status/QoL, five functional scales (physical, social, cognitive, role, and emotional), three symptom scales (pain, fatigue, and nausea and vomiting) and six symptom items (diarrhoea, insomnia, constipation, appetite loss, dyspnoea, and financial difficulties) [2]. This questionnaire is often completed by participants multiple times throughout a study to enable assessment of the change in QoL over time.

Several statistical challenges are encountered when analysing longitudinal QoL data. Firstly, missing data is a common problem when assessing QoL over time, with missing observations a frequent occurrence [3]. This is even more of a problem in cancer studies where missing data due to sickness and/or death are more likely to occur. Secondly, repeated measurements of QoL from the same individual result in a nested data structure; the repeated measurements are highly dependent and likely to be more correlated than those from different individuals [4]. Nested data structures violate the independence assumption, a key assumption of traditional statistical methods such as multivariable regression and analysis of variance (ANOVA) [3, 5, 7]. Another challenge, particularly relevant in cancer studies, is that longitudinal QoL data and survival data are often related in some way, with the time to event (e.g., disease progression or death) potentially associated with the QoL trajectory [8]. If this is the case, separate analyses of QoL and survival data may lead to biased and less precise results by not accounting for this possible association [8, 10].

Specific challenges related to the QLQ-C30 questionnaire include the multinomial nature of symptom items, such as appetite loss, where the score can only take four possible values. Questions arise over whether these scores should be treated as continuous outcomes, or if they should be considered categorical/ordinal. Another issue that arises is the occurrence of floor/ceiling effects, where a high proportion of participants experience perfect functioning (highest possible score) or no symptoms (lowest possible score), resulting in a large peak at one end of the score range. The resulting distribution can lead to usual analysis methods, such as the linear mixed effects model, having an inadequate fit. The challenge is to find an analysis method that provides an acceptable fit to the data whilst providing results that are easy to interpret and clinically relevant.

It is recognised that analysis methods that account for the multilevel structure of longitudinal QoL data are the recommended approach to reduce the likelihood of type I errors (i.e. a false positive result), biased parameter estimates and incorrect inferences being made [5, 11, 12]. However, many multilevel modelling methods are available. Methods that model the QoL score as a continuous outcome include linear mixed effects models, generalized estimating equations (GEEs), pattern mixture models (PMMs), and joint longitudinal survival models. These methods have different underlying assumptions, including those regarding the missing data mechanism, and account for the correlation between repeated observations within a participant in different ways [4, 9, 10, 13, 17].

Two-part models, or mixed distribution models, can be used when the outcome variable has a mixed distribution and thus are a possible approach if the QoL score exhibits a floor or ceiling effect [4]. However, the interpretation of the two-part model is difficult and may lead to researchers choosing a simpler analytical approach, such as categorising the score and performing ordinal or logistic regression.

The different statistical methods that can be used to analyse QoL over time all provide different effect estimates, levels of precision, bias, and P values, and potentially different inferences being drawn. Whilst methods exist to handle problems such as missing data due to sickness/death, the ordinal nature of symptom items, and other issues described above, it is unknown how often these are implemented in practice.

In February 2020 the Setting International Standards in Analyzing Patient-Reported Outcomes and Quality of Life Endpoints Data for Cancer Clinical Trials (SISAQOL) consortium published recommended methods for the statistical analysis of QoL data for a number of outcome types [18, 19]. Essential and desirable criterion used by the consortium to evaluate different statistical models included: perform a statistical test between treatment groups; produce clinically relevant results; adjust for covariates, including the baseline QoL score; handle clustered data; and handle missing data with the least restrictions.

The aim of this paper was to comprehensively describe the types of statistical methods used in practice when analysing QoL scores derived from the QLQ-C30 questionnaire, to explore potential patterns relating to the type of study and methods used, and examine whether the analysis method was consistent with the stated estimand for studies using QoL as the primary outcome. The focus was on randomised controlled trials (RCTs) and prospective observational studies published in the literature. The scoping review methodology was chosen due to the expansive inclusion criteria and broad nature of the proposed topic.

## Methods

### Study design

A protocol for this scoping review was drafted a priori using the JBI template for scoping reviews [20]. The protocol was finalised on 16 February 2023 and was subsequently made publicly available [21]. The JBI Manual for Evidence Synthesis and Preferred Reporting Items for Systematic Reviews and Meta-Analysis Extension for Scoping Reviews (PRISMA-ScR) statement were used to guide the conduct and reporting of this review [22, 23]. The PRISMA-ScR checklist can be found in supplementary appendix 1.

### Eligibility criteria

The full eligibility criteria were stipulated a priori. To be included in the review, articles needed to be RCTs or prospective observational studies that assessed QoL using the EORTC QLQ-C30 questionnaire with or without the complementary QLQ-LC13 questionnaire. The studies needed to collect QoL at two or more post-baseline/randomisation time points as the focus of this review is on statistical methods used to assess changes in QoL over time. The QoL scores derived from the questionnaires had to be treated as the outcome measure and be analysed statistically (i.e., analysed using a statistical model). Only studies published in English between 1 January 2021 and 31 December 2022 were included to capture recent statistical methods used.

Cross-sectional and descriptive study designs such as case series were not included. Meta-analyses (unless using individual patient data), text and opinion papers, conference abstracts, and clinical trial protocols were also excluded. Studies that did not report the statistical methods used for the QoL analyses were excluded, as the aim of the review was to identify the types of statistical methods used to analyse these data. No restriction was placed on the number of participants included in each study or the journal where the article was published.

### Information sources and search

To identify potentially relevant articles, MEDLINE and Embase databases accessed via OvidSP were searched. The final Embase search strategy can be found in supplementary appendix 2. An academic medical subject librarian from the University of Bristol reviewed the search strategy. Sources of unpublished studies/grey literature were not searched as the review focused on statistical methods used in published studies. Initial searches of MEDLINE and Embase databases were first performed on 1 December 2022 so the full search strategy could be included in the protocol. The search strategy was simplified and re-ran on 16 February 2023, to capture any articles published in December 2022. Any articles appearing in the first search but not the second were excluded. Reasons why articles no longer appeared in the newer search were identified.

### Study selection

Following the search, all identified citations were collated, exported into EndNote X9.3.3 and duplicates were removed. Titles and abstracts were screened by one author (RAH) for assessment against the inclusion criteria. Potentially relevant sources were retrieved in full. One author (RAH) screened all full text articles, and a subset of randomly selected citations were assessed in detail against the inclusion criteria by three independent reviewers (JH, CAR, RE) and agreement between reviewers compared. Disagreements between the reviewers on study selection were resolved through discussion.

### Data extraction

A REDCap database was developed, and pilot tested by one author (RAH) who modified and revised the data items collected and subsequently extracted data from all eligible papers. Two independent reviewers (JH, RE) each extracted a random subset of eligible papers to ensure consistency in the data extracted. Again, any disagreements were resolved through discussion between reviewers.

Extracted data related to the study design and characteristics included: study design, questionnaires used, whether QoL was completed at baseline, frequency of follow up (excluding baseline), whether any QoL score was the primary outcome and any associated sample size details, minimal clinically important differences (MCIDs), and overall sample size. Data related to the statistical analyses included: scores analysed, methods to account for missing questionnaires and missing data due to death, statistical models used, time points analysed and included in each model, form of the QoL score modelled (i.e. raw score, change from baseline), covariates included in the models, how time was fitted in the models (categorical or continuous), whether effect sizes and 95% confidence intervals (95% CIs) were presented, and whether P values were presented. See supplementary appendix 3 for the data collection form.

### Synthesis of results

Extracted data were summarised using descriptive statistics. Continuous data were summarised by the mean (standard deviation) or median (interquartile range [IQR]) if distributions were skewed, and categorical data are reported as a number and percentage. Analysis methods used in the included studies are described overall and by type (RCT vs other). Reported studies that were from a single RCT of any design or included data from multiple RCTs were classified as RCT. All remaining articles were classified as other (e.g., cohort studies or studies pooling data from RCTs and non-RCTs). The statistical analysis of a QoL primary outcome was considered appropriate for the specified estimand if the method chosen was appropriate for the QoL outcome defined (e.g. global health status score analysed using a linear mixed model) and the analysis was based on the time frame specified (e.g., specified 12 months and reported results at 12 month time point). If a specific QoL outcome was not stated, the analysis was considered appropriate if the method of analysis was appropriate for the QoL outcomes analysed (e.g. ‘QoL’ specified as primary outcome and analysed using multiple t-tests). If a relevant time frame was not specified, the analysis was deemed appropriate if all time points were analysed. Data management and analysis was performed in Stata version 18.0 (StataCorp, College Station, TX).

## Results

### Study selection

**Fig. 1:**
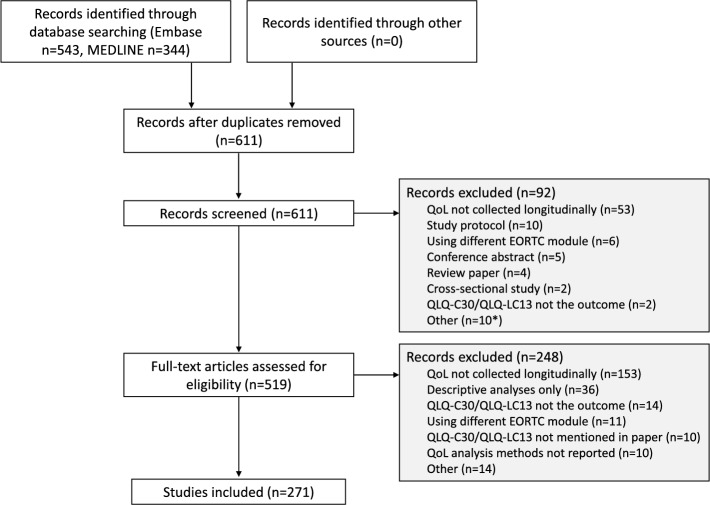
Flow diagram of study selection. * including one instance where the full-text article could not be obtained and so the record was excluded

The search returned 543 records from the Embase database and 344 from the MEDLINE database (Fig. 1); 611 records remained for title and abstract screening after removing duplicates. Following this, 92 articles were excluded for reasons detailed in Fig. 1, leaving 519 records for full text screening. The full text could not be retrieved for one article and so this study was excluded. The main reasons articles were excluded at the full text screening stage were because QoL was not collected longitudinally (*n* = 153), no formal statistical analysis was performed (n = 36), or the QoL was not treated as the outcome measure (*n* = 14). A total of 271 articles met the eligibility criteria and were included in the review. Due to limited available resources, a pragmatic decision was made to initially select 15 articles to be second screened by three reviewers (including two reviewers screening the same five articles); discrepancies were found in two instances and were resolved, with the second reviewer agreeing with the original decision. Similarly, data were extracted from a further 15 articles by two reviewers (including both reviewers extracting the same five articles); discrepancies were found in < 6% of data points collected (one question = one data point). Discrepancies were the result of the clarity of reporting or misreading of articles and not due to a difference in interpretation of methods used. Again, after discussion, the second reviewer agreed with the original extraction. Based on the level of agreement observed and given the reviewers all agreed with the original screening/extraction, the team felt there was no need for further double screening or extraction.

Fifty-six records appeared in the initial search but were not identified in the final search due to the database record being updated in the interim period: thirty-five of these records did not have either of the QLQ-C30 or QLQ-LC13 questionnaires indexed or mentioned in the title or abstract; nine did not have the required wording/indexing to identify them as an RCT or observational study; seven were classified as conference abstracts; four had the date of publication updated to 2023; and one record was removed from both MEDLINE and Embase databases.

### Study characteristics

The characteristics of the eligible studies included in the review are presented in Table 1. The majority of included studies were parallel group RCTs (161/271, 59%), followed by cohort studies (84/271, 31%). The remaining studies were other RCT designs, single arm trials, or used individual patient data from multiple RCTs, multiple cohort studies, or combined data from cohorts and RCTs. The median number of patients included in each study was 201 (IQR 90 to 508). Quality of life was assessed at baseline in 96% (261/271) of included studies, and the median number of post-baseline/randomisation assessments was 4 (IQR 3 to 6). Most studies used just the QLQ-C30 questionnaire, none used just the QLQ-LC13 questionnaire, and 16 studies used both. In all cases the analysis method for the two questionnaires was the same. Therefore, without loss of generality, the results that follow refer to the QLQ-C30 questionnaire only. Very few studies specified a primary outcome that was a QoL score derived from the QLQ-C30 questionnaire (34/271, 13%). Of the 271 studies included in the review, 131 (48%) defined an MCID; the majority of these stated an MCID that related to within-patient change in QoL score (84/131, 64%), 28% (37/131) defined an MCID representing a between-group difference, and 8% (10/131) specified MCIDs for both within-patient changes and between-group differences.

**Table 1:** Characteristics of studies included in review

Study characteristics	Number of studies (n = 271)
Study design	
Parallel group RCT	161/271 (59%)
Cohort study	84/271 (31%)
Data from multiple RCTs	7/271 (3%)
Data from multiple cohorts	4/271 (1%)
Pooled cohort from RCT	4/271 (1%)
Other	11/271 (4%)
Number of patients included in study*	201 (90, 508)
Questionnaires used	
QLQ-C30	255/271 (94%)
QLQ-C30 and QLQ-LC13	16/271 (6%)
Assessment time points	
QoL assessed at baseline	261/271 (96%)
Total post baseline/randomisation assessments^	4 (3, 6)
Primary outcome	
QoL primary outcome	34/271 (13%)
Specific domain chosen—QoL score analysed	15/34 (44%)
Specific domain chosen—outcome derived from QoL score analysed (e.g., proportion improving by MCID)	3/34 (9%)
Specific QoL domain not specified (e.g., HRQoL)	16/34 (47%)
MCID	
MCID defined	131/271 (48%)
Type of MCID defined	
Within-patient change	84/131 (64%)
Between-group difference	37/131 (28%)
Within-patient change and between-group difference	10/131 (8%)

Data are presented as n/N (%) or median (IQR). *RCT* randomised controlled trial, *QLQ-C30* Quality of Life Questionnaire Core 30, *QoL* quality of life, *MCID* minimal clinically important difference

^*^ Number of patients randomised if RCT or number enrolled/included if observational study

^ Total number of post baseline/randomisation assessments could not be determined for 27 studies; 15 studies collected QoL continuously during treatment/until disease progression/until death/until study cut-off date, six had a different number of assessments per group, four had a different number of assessments per cohort/trial, one had a different number of assessments per questionnaire and one did not provide enough information

### Synthesis of results

Just over half of studies (138/271, 51%) analysed all scores derived from the QLQ-C30 questionnaire, 28% (76/271) selected a subset of scores and 4% (11/271) only analysed summary scores (Table 2). Around three-quarters of studies (207/271, 76%) did not use any methods to account for missing questionnaires or state the assumed missing data mechanism. Only 23 studies (8%) explicitly used a method to account for missing data due to death, such as joint modelling of the QoL score and survival or defining death as an event in a time to event (TTE) analysis. The remaining studies treated data truncated by death in the same way as other missing data.

**Table 2:** Analysis methods by type of study

	**RCT (n = 172)**	**Other (n = 99)**	**Overall (n = 271)**
Scores analysed			
All scores	86/172 (50%)	52/99 (53%)	138/271 (51%)
Subset of scores	49/172 (28%)	27/99 (27%)	76/271 (28%)
Subset of scores plus summary score	24/172 (14%)	7/99 (7%)	31/271 (11%)
All scores plus summary score	8/172 (5%)	7/99 (7%)	15/271 (6%)
Only summary scores	5/172 (3%)	6/99 (6%)	11/271 (4%)
Analysis methods			
Number of statistical methods used			
1	88/172 (51%)	63/99 (64%)	151/271 (56%)
2	66/172 (38%)	29/99 (29%)	95/271 (35%)
> 2	18/172 (10%)	7/99 (7%)	25/271 (9%)
Any method used to account for data truncated by death	18/172 (10%)	5/99 (5%)	23/271 (8%)
Methods to account for questionnaires missing for other reasons			
Yes – applied specific method e.g. multiple imputation	25/172 (15%)	7/99 (7%)	32/271 (12%)
No – stated assumed MAR/MCAR	21/172 (12%)	11/99 (11%)	32/271 (12%)
No	126/172 (73%)	81/99 (82%)	207/271 (76%)
Statistical model used			
Linear mixed effects model	81/172 (47%)	40/99 (40%)	121/271 (45%)
Time to event analysis	49/172 (28%)	5/99 (5%)	54/271 (20%)
T-test	29/172 (17%)	15/99 (15%)	44/271 (16%)
Mann–Whitney U/Wilcoxon rank-sum test	25/172 (15%)	13/99 (13%)	38/271 (14%)
Any longitudinal analysis method*	122/172 (71%)	67/99 (68%)	189/271 (70%)
Multiple cross-sectional analyses	58/172 (34%)	40/99 (40%)	98/271 (36%)
Adjustment for multiplicity	7/58 (12%)	3/40 (8%)	10/98 (10%)
Longitudinal and cross-sectional analyses ×	22/172 (13%)	16/99 (16%)	38/271 (14%)
Estimated treatment effect at multiple time points from longitudinal analysis model ×			
Yes	29/111 (26%)	11/64 (17%)	40/175 (23%)
Yes, if time*treatment interaction significant	3/111 (3%)	0/64 (0%)	3/175 (2%)
Adjustment for covariates/confounding^			
All models	52/172 (30%)	32/99 (32%)	84/271 (31%)
Some models	23/172 (13%)	18/99 (18%)	41/271 (15%)
No models	86/172 (50%)	44/99 (44%)	130/271 (48%)
Unclear	11/172 (6%)	5/99 (5%)	16/271 (6%)
Effect size and 95% CI/SE presented from at least one model	99/172 (58%)	53/99 (54%)	152/271 (56%)
Effect size and 95% CI/SE presented			
All models	69/172 (40%)	40/99 (40%)	109/271 (40%)
Some models	30/172 (17%)	13/99 (13%)	43/271 (16%)
No models	73/172 (42%)	46/99 (47%)	119/271 (44%)

Data are n/N (%). *RCT* randomised controlled trial, *MAR* missing at random, *MCAR* missing completely at random, *CI* confidence interval, *SE* standard error. Four of the 99 studies classified as other combined data from RCTs and observations studies

^*^ includes time to event analyses. ^ other than treatment group, time, and their interaction if applicable. × excludes time to event analyses

**Fig. 2:**
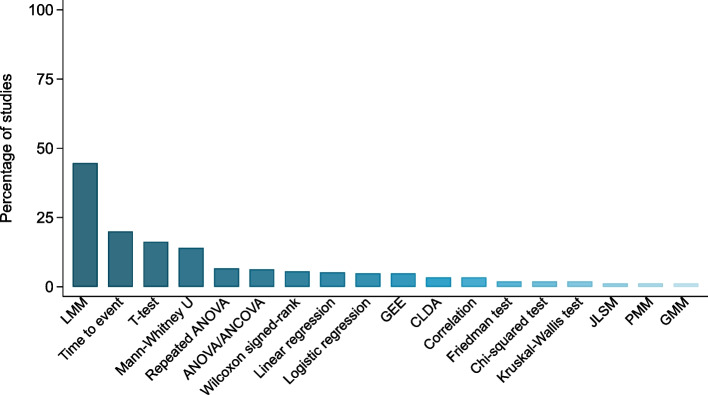
Analysis methods used in included studies. Method presented if used in > 1% of studies reviewed. LMM = linear mixed model, CLDA = constrained longitudinal data analysis, PMM = pattern mixture model, GEE = generalised estimating equations, JLSM = joint longitudinal survival model, GMM = growth mixture modelling

Overall, the most utilized statistical model was a linear mixed effects model, with 45% (121/271) of included studies applying this approach in at least one analysis (Fig. 2). Following this, the models/methods used most often were TTE analyses (54/271, 20%), t-tests (44/271, 16%) and Mann–Whitney U/Wilcoxon rank-sum tests (38/271, 14%). Thirteen studies used unweighted GEEs (5%), nine used constrained longitudinal data analysis (3%), three used growth mixture models (1%), three used PMM (1%) and three used joint longitudinal survival models (1%). Just over two-thirds (189/271, 70%) of studies applied at least one longitudinal analysis method. Thirty-eight studies performed both longitudinal and cross-sectional analyses. Multiple cross-sectional analyses were undertaken in 98 studies (36%), and of these, adjustment for multiplicity was made in 10% (10/98). The most common method of multiplicity adjustment was the Bonferroni correction. Of the 175 studies that applied at least one longitudinal analysis model (excluding TTE analyses), 40 (23%) estimated treatment effects at multiple time points regardless of the statistical significance of the time x treatment interaction; 3/175 estimated treatment effects at multiple time points only if the time x treatment interaction was statistically significant (Table 2).

Table 2 summarises analysis methods by study type (RCT vs other). One-hundred and seventy-two studies were classified as RCTs and 99 were classified as other. Linear mixed effect models were used slightly more frequently in RCTs (81/172, 47%) compared to other studies (40/99, 40%). Similarly, TTE analyses, such as time to deterioration in QoL score by the MCID, were also more likely to be performed in RCTs compared to other studies, with 28% (49/172) of RCTs included in the review using this method, compared to 5% (5/99) of other studies. The use of the other commonly applied statistical methods (e.g., t-tests and Mann–Whitney U/Wilcoxon rank-sum tests) was comparable between RCTs and other studies.

Covariates or confounding variables, such as the baseline QoL score, were accounted for in at least one QoL analysis in 47% (75/161) of RCTs and 53% (50/94) of other studies (Table 2). Effect sizes and 95% CIs or standard errors (SEs) from at least one statistical model were presented in 56% of studies included in the review (99/172, 58% of RCTs vs 53/99, 54% of other studies). Overall, 40% of RCTs and other studies presented effect sizes and a measure of precision from all statistical models; 44% of studies did not present this information for any statistical analysis presented, either because the output from the chosen analysis did not include this information, or the authors chose not to present it.

Further details about the analyses performed are presented in Table 3. The majority of studies (248/270, 92%) analysed or used data from all time points collected, either in one or separate analyses. Most studies used the QoL score as the outcome measure (216/271, 80%), followed by change from baseline (56/271, 21%). Of those applying a longitudinal method of analysis (excluding TTE analyses), the majority fitted time as a categorical variable as recommended by the SISAQOL consortium (150/168, 89%). Of the 54 using TTE analyses, 16 (30%) used Cox proportional hazards models to estimate the difference between groups, compared to 32 (59%) using the log-rank test.

**Table 3:** Additional details

Additional details	Number of studies (n = 271)
Analysed/used data from all time points collected^	248/271 (92%)
Form of QoL score modelled*	
Raw score	216/271 (80%)
Change from baseline	56/271 (21%)
Proportion achieving MCID	8/271 (3%)
Other	82/271 (30%)
For longitudinal analysis methods, time fitted as categorical or continuous* ×	
Categorical	150/168 (89%)
Continuous	20/168 (12%)
Discussed checking model assumptions/fit of residuals	
Yes	36/271 (13%)
No	197/271 (73%)
N/A, used methods that did not require assumptions to be checked e.g. Mann–Whitney U test	38/271 (14%)
Analysed single item score ±	165/267 (62%)
Used method to account for ordinal nature of single item score	33/158 (21%)
Method used*	
Non-parametric	26/33 (79%)
Logistic regression	5/33 (15%)
Ordinal logistic regression	2/33 (6%)
Two-part model	1/33 (3%)
Multinomial regression	1/33 (3%)
Analysis method that would account for a peak in QoL score at end of distribution	
Yes	40/266 (15%)
No	116/266 (44%)
NA, did not analyse outcome that would be affected by peak	110/266 (41%)
Method used*	
Non-parametric	30/40 (75%)
Logistic regression	4/40 (10%)
Ordinal logistic regression	3/40 (8%)
Two-part model	2/40 (5%)
GEE Tweedie model	1/40 (3%)
Other	2/40 (5%)
Any time to event analysis	54/271 (20%)
Survival analysis methods used	
Kaplan–Meier methods, Cox proportional hazards models and log-rank test	23/54 (43%)
Kaplan–Meier methods and Cox proportional hazards models	15/54 (28%)
Kaplan–Meier methods and log-rank test	8/54 (15%)
Kaplan–Meier methods only	6/54 (11%)
Cox proportional hazards model and log-rank test	1/54 (2%)
Cox proportional hazards model only	1/54 (2%)
Cited SISAQOL recommendations	10/271 (4%)
Used approach recommended by SISAQOL	
Yes	73/255 (29%)
Partly, for some analyses but not all	44/255 (17%)
No	138/255 (54%)

Data are n/N (%). * not mutually exclusive. ^ either in one or separate analyses. × excluding TTE analyses. ± single item scores only take four possible values

In relation to the specific challenges faced when analysing QLQ-C30, 165/267 (62%) of studies analysed a single item symptom score (a score that takes only four possible values, Table 3). Of these, 21% (33/158) used a method of analysis that accounted for the ordinal nature of the single item score. The remaining treated the score in the same way as other functioning or symptom scales analysed. Methods used included: non-parametric methods (26/33, 79%), logistic regression (5/33, 15%), ordinal logistic regression (2/33, 6%) and two-part models (1/33, 3%). Similar approaches were used to account for a possible peak at one end of the score distribution; methods used included non-parametric methods, ordinal logistic and logistic regression, two-part models, and GEE Tweedie models (Table 3). Few studies mentioned checking of the assumptions of their chosen analysis method and/or examining model fit (36/233, 15%).

Ten of the included studies cited the SISAQOL guidelines; 255/271 analysed at least one outcome that the SISAQOL consortium had published a recommended analysis method for. Just under one third of studies (73/255, 29%) followed the recommended approach for all outcomes, 17% (44/255) followed recommendations for some outcomes but not all, and 54% (138/255) did not follow the recommended analysis methods.

Of the 34 studies (17 RCTs, 17 other designs) that used QoL as a primary outcome, only 14/34 (41%) studies (7 RCTS, 7 other designs) clearly defined the QoL outcome of interest e.g. QLQ-C30 fatigue or global health status, and the relevant time frame (Table 4). The statistical analysis was appropriate for the specified estimand in 27/34 (79%) studies (13 RCTS, 14 other designs) and 15 of these 27 studies reported an effect estimate and measure of precision (5 RCTS, 10 other designs). Twenty-three studies reported deaths; nine included patients who died in their analyses, nine performed cross-sectional analyses and so excluded deaths if they occurred prior to the time point(s) analysed, and five excluded deaths from all primary outcome analyses.

**Table 4:** Studies with QoL as the primary outcome and estimand details

Ref	Study type	Outcome defined in estimand	Time frame	Statistical analysis appropriate for estimand	If no, why not	Effect estimate presented	Confidence interval/standard error presented	Deaths reported during study	If yes, how were they handled in primary outcome analysis
2	RCT	Change in QLQ-C30 physical function	Over 1 year post-randomisation	No	Averaged scores over first 4 years	Yes	Yes	Yes, 20%	Complete case analysis—excluded all deaths
9	Other	QLQ-C30 GHS	Not specified	Yes	-	Yes	Yes	Yes, 23%	Patients included up to the point of death
17	RCT	Change since baseline in score representing problem prioritised by patient or GHS if none specified	Over 12 weeks	Yes	-	Yes	Yes	Yes, < 5%	Modified ITT – excluded all deaths
18	RCT	QoL	Not specified	Yes	-	Yes	Yes	No	-
19	RCT	QLQ-C30 summary score	At 12 months	Yes	-	Yes	Yes	Yes, % not reported	Patients included up to the point of death. Sensitivity excluding deaths before 12 months
23	RCT	QLQ-C30 GHS	Not specified	Yes	-	No	No	Yes, 47%	Patients included up to the point of death
25	RCT	QoL	Over 3 months post-surgery	Yes	-	No	No	No	-
34	Other	Mean QoL score	At 1, 1.5 and 2 years post-surgery	Yes	-	Yes	Yes	Yes, 16%	Patients included up to the point of death
42	Other	Difference in QoL between groups	At 12 months post-treatment	Yes	-	Yes	Yes	No	-
50	Other	Good QLQ-C30 GHS (≥ 70) at any point	Any point during follow up	No	Presented predictors of decreased GHS, not good GHS	Yes, but HR from logistic regression not RR/OR	Yes	No	-
63	RCT	QLQ-C30 GHS	Not specified	Yes	-	No	No	No	-
76	Other	QoL compared to reference population	Before, during and after treatment	No	Did not test QoL compared to reference population	No	No	Yes, % not reported	Cross-sectional analyses, complete case – deaths excluded if died before time points analysed
78	RCT	QLQ-C30 summary score	Over 5 months	Yes	-	No	No	Yes, 7%	Cross-sectional analyses, complete case – deaths excluded if died before time points analysed
83	RCT	Change in QLQ-C30 GHS	At 1 week after treatment	Yes	-	No	No	Yes, % not reported	Complete case analysis—excluded all deaths
84	Other	QoL	At 3 years after surgery	Yes	-	Yes	Yes	Yes, 12%	Patients included up to the point of death
95	RCT	QLQ-C30 summary score	Not specified	Partly	Did not specify time frame and did not analyse all time points	No	No	No	-
99	RCT	Improvement in QoL	At day 42	No	No definition of improvement, just analysed raw score	No	No	No, eligibility criteria included life expectancy > 3 months, last follow up time point 84 days	-
100	Other	Good QLQ-C30 GHS (≥ 70) at any point	Any point during follow up	Yes	-	Yes	Yes	No	-
115	RCT	QoL	Baseline to 12 weeks	Yes	-	No	No	No	-
118	Other	QLQ-C30	Not specified	Partly	Did not specify time frame and did not analyse all time points	No	No	Yes, 41%	Cross-sectional analyses, complete case – deaths excluded if died before time points analysed
123	Other	QLQ-C30 fatigue	At 1 year post-surgery	Yes	-	Yes	Yes	Yes, % not reported	Cross-sectional analysis, complete case – deaths excluded if died before 1 year
124	Other	QLQ-C30 fatigue	At 1, 1.5 and 2 years post-surgery	Yes	-	Yes	Yes	Yes, 10% died within two months of response	Patients included up to the point of death. Sensitivity excluding deaths within 2 months of response
129	Other	Severe QLQ-C30 fatigue (≥ 40)	At 1, 2 and 4 years post-diagnosis	Yes	-	Yes	Yes	Yes, % not reported	Cross-sectional analyses, complete case – deaths excluded if died before time points analysed
134	RCT	QoL	During treatment	Yes	-	No	No	Yes, < 5%	Cross-sectional analyses, complete case – deaths excluded if died before time points analysed
139	RCT	QoL	At 12 months	No	Reported between group difference over 12 months not at 12 months	No	No	Yes, 36%	Complete case analysis—excluded all deaths
159	Other	Change in QoL	Baseline to follow up	Yes	-	Yes	Yes	No	-
171	Other	Difference in QoL	During follow up	Yes	-	Yes	Yes	Yes, 82%	JLSM with treatment failure as survival event
180	Other	QoL	Not specified	Yes	-	Yes	No	Yes, 79%	Cross-sectional analyses, complete case – deaths excluded if died before time points analysed
191	RCT	Delay in time to deterioration for each PRO measure	Time to deterioration by MCID	Yes	-	Yes	Yes	Yes, % not reported	Deaths included if died after first follow up. Sensitivity including death as an event
198	Other	Change in QLQ-C30 GHS	Following surgery	Yes	-	No	No	Yes, % not reported	Complete case analysis—excluded all deaths
213	Other	QoL	All post-op time points	Yes	-	Yes	No	Yes, % not reported	Cross-sectional analyses, complete case – deaths excluded if died before time point analysed
236	RCT	QLQ-C30 GHS	At 6 months	Yes	-	No	No	No	-
241	RCT	QLQ-C30 physical functioning	At 5 weeks	Yes	-	Yes	Yes	Yes, 6%	JLSM with death as survival event
247	Other	QLQ-C30 GHS	At 24 months	Yes	-	No	No	Yes, 3%	Cross-sectional analysis, complete case – deaths excluded if died before 24 months

*QoL* quality of life, *GHS* global health status, *JLSM* joint longitudinal survival model, *MCID* minimal clinically important difference**.** References can be found in Supplementary Appendix 4

## Discussion

This scoping review examined the statistical methods applied to analyse longitudinal QLQ-C30 scores in 271 RCTs and prospective observational studies published in 2021 and 2022. The review demonstrated that there is no current consensus regarding the statistical approach to use when analysing longitudinal QoL data. More than 18 different statistical methods were applied in the studies reviewed, with the most frequently used model, a linear mixed effects model, being used in less than half of the studies.

Despite multilevel/repeated measures regression models being the recommended approach to analysing longitudinal QoL data, and the handling of clustered data deemed a desirable criterion by the SISAQOL consortium, approximately one third of studies included in this review did not use any statistical analysis method that took the longitudinal structure of their data into account, opting for cross-sectional comparisons (e.g. t-tests, Mann–Whitney U/Wilcoxon rank-sum tests, ANOVA, chi-squared tests) instead [5, 19]. Cross-sectional comparison techniques do not take into account the correlation between repeated measurements and unless a clinically defined a priori timepoint was specified, there is the potential for inferences to vary according to (an arbitrary) choice of timepoint and the possibility that true treatment effects are missed. Ignoring the correlation between repeated measurements may lead to biased, less precise estimates of treatment effects and potentially inappropriate scientific conclusions being drawn [6, 7, 11, 12, 24]. Performing multiple cross-sectional analyses greatly increases the risk of a false positive result (increased type I error) unless adjustments for multiplicity are made. This review found around one third of studies performed multiple cross-sectional analyses without any adjustment for multiplicity. The issue of multiplicity should be considered whenever multiple time points are of interest, as presenting treatment effect estimates and p-values from multiple time points from a longitudinal analysis increases the risk of type I error. Just under a quarter of studies who performed a longitudinal analysis estimated treatment effects at multiple time points irrespective of the statistical significance of the time x treatment interaction. Whilst adjustments for multiplicity reduce the likelihood of a type I error, they are often conservative and lead to a reduction in power resulting in a less efficient statistical analysis [25, 27]. One possible solution to minimise the risk of a type I error is to fit a longitudinal model (e.g. linear mixed effects model) with a time x treatment interaction term and if this is not statistically significant at a pre-specified significance level (e.g. 10% significance), then an overall treatment effect can be reported with justification that there was no evidence to suggest that the treatment effect changed over time. If the time x treatment interaction is found to be statistically significant, then an effect estimate and measure of precision at each time point can be reported. However, this approach is rarely used in practice, with only three studies following this analysis strategy. Another limitation of applying cross-sectional analysis methods is the increased risk of biases when missing data are present [12]. As cross-sectional analysis methods, such as linear regression and t-tests, only use complete cases, analyses can result in biased estimates unless the missing data mechanism is missing completely as random, an unlikely occurrence when assessing QoL in cancer clinical trials [28, 30]. In addition, the advantage of collecting QoL data over time is the ability to directly assess the change over time within and between individuals, as well as increasing precision and power to detect treatment effects, particularly as the correlation between time points increases [12, 31, 32]. By not applying longitudinal analysis methods, researchers are not making the most of the data available and are potentially wasting the time and resources used to collect these data in the first place.

Less than half of studies with QoL as the primary outcome clearly defined the estimand of interest leading to potential ambiguity in the interpretation of the studies’ results. The use of the estimands framework for QoL outcomes has been discussed in detail in the literature, and highlights the importance of a clearly defined estimand to enable clear interpretation of QoL outcomes [33, 36]. Lawrance et al. discuss the estimand framework and provide a useful example of how to build an estimand for a QoL objective [35].

A very small proportion of studies employed methods that accounted for missing data due to disease progression/death, a common problem in cancer studies. The most common way of adjusting was in TTE analyses, by treating disease progression or death as an event or competing risk. A comprehensive discussion of TTE methods for QoL outcomes has been undertaken [37]. Although discussed in detail in the literature, only 1% of studies used a joint longitudinal survival model and 1% used pattern mixture models, perhaps due to a lack of understanding of how to implement these methods in practice [4, 9, 10, 16, 38, 40]. Another reason for not explicitly accounting for data truncated due to death could be that researchers believe the missing data are non-informative and the MAR assumption of linear mixed models, for example, holds. Fairclough et al. demonstrated that in scenarios with higher proportions of death and missing data due to disease progression, analysis methods are sensitive to the underlying model assumptions and the MAR assumption may not be justified [28]. Joint longitudinal survival models can make use of additional information relating to time to disease progression/death which results in more precise estimates. It is recommended to consider data truncated by death differently to missing data due to other reasons and carefully consider the research question and different methodological approaches available [30, 41, 44].

Both CONSORT and STROBE reporting guidelines highlight the importance of providing an estimate of precision, such as a 95% CI or SE, for each estimated treatment effect, and the SISAQOL consortium state producing clinically relevant results as an essential criterion [19, 45, 46]. This review found that only 41% of studies provided an effect size and measure of precision for every statistical model fitted, and 44% did not report an estimate of precision for any analyses completed, often solely reporting P values. Whilst the P value demonstrates whether a statistical difference exists, it does not indicate the magnitude of the effect nor the certainty surrounding the estimate, both of which are essential for clinicians to be able to judge the relevance of any difference between groups. Fewer than 50% of studies defined an MCID upon which to assess either the change in QoL within patients or the clinical relevance of the between-group effect sizes observed. Whilst assessing the statistical properties of results is important, it is essential to also evaluate the clinical relevance of findings, as a statistically significant finding may not be a clinically important finding. There was also variation in the MCIDs chosen for those studies that did define one, with some specifying different MCIDs for different subscale scores whereas others applied the same MCID for all scores. A potential reason for this variation in MCIDs could be that several different MCIDs for the QLQ-C30 scores have been proposed and some may be more applicable to certain patient groups [47, 52].

Another desirable criterion stated by the SISAQOL consortium is to adjust for baseline covariates, including the baseline QoL score [19]. As the baseline QoL score is highly likely to be correlated with QoL outcome, adjusting for the baseline score will lead to a more efficient analysis with respect to the precision of the effect estimate. They also stated it is important to adjust for other prognostic covariates such as disease characteristics, demography, and site. Around 50% of the studies included in this review did not perform any adjustment for baseline covariates, despite the majority of studies collecting baseline QoL and demographic details, meaning the statistical analyses are not as efficient as they could be.

Regarding the analyses of single item symptom scores, most studies that analysed these outcomes treated the score as continuous rather than categorical or ordinal. The advantage of this approach is that the effect estimate, a mean difference, is easier to interpret, especially in terms of clinical significance where the majority of MCIDs are defined on a continuous scale. This is likely the reason that researchers opt for this analytical approach for these outcomes. Non-parametric methods have the advantage of making no distributional assumptions, so are useful for ordinal scores or scores exhibiting peaks at one end of the distribution; this review found these are most likely to be used as an alternative analysis method as they have the disadvantage of not providing effect estimates upon which to judge the clinical significance. Two-part models, ordinal logistic and logistic regression have the advantage that they do provide effect estimates and measures of precision, but the resulting outcome measure is more challenging to interpret.

This review focussed on studies using the EORTC QLQ-C30/QLQ-LC13 questionnaires; however, the findings are likely to apply to other longitudinal measurements, with similar statistical challenges being faced when analysing any longitudinal data. As this review did not restrict included articles to specific journals or study designs, a wide variety of studies were included, with varying degrees of quality in both the methods applied and how the analyses were reported. The advantage of this was it resulted in a broad picture of the statistical methods applied in the literature, highlighting the significant variation in approaches. However, in some studies it was hard to determine every detail of the methods applied, particularly in terms of how the models were specified meaning some aspects had to be classified as unclear.

The main limitation of this review is that some relevant sources of evidence will have been missed due to only searching two databases and these databases did not offer full text searching. The QLQ-C30/QLQ-LC13 are often secondary outcomes in studies and thus may not be mentioned in the abstract or chosen as a keyword by the authors. With MEDLINE and Embase databases performing automatic indexing (with some manual indexing for selected articles), it is likely some articles will not have had the questionnaire names indexed and so the study will not have appeared in the search results [53, 54]. Although some sources of evidence will have been missed, the aim of this scoping review was to comprehensively describe the statistical methods used to analyse these QoL data and given vast heterogeneity found in the methods used, it is unlikely that the statistical methods applied in the studies that were missed will differ from those included in the review. The SISAQOL guidelines were only published 11 months prior to the start of the two-year inclusion period for this review and so there may not have been sufficient time for the guidelines to be implemented by researchers. The guidelines are also for RCTs, and this review included non-RCTs, although one cohort study did reference the guidelines indicating researchers may follow the recommended approaches irrespective of study design.

## Conclusions

The QLQ-C30/QLQ-LC13 questionnaires are often used in cancer studies to assess change in QoL over time. This review found that methods used in practice vary considerably, with many not following the recommended approaches to analyses and presentation of results. Methods that ignore the longitudinal nature of the data are still frequently used in practice. A third of studies performed multiple cross-sectional analyses without any adjustment for multiplicity, a quarter estimated treatment effects from longitudinal models at multiple time points irrespective of the statistical significance of the time x treatment interaction, and over 40% failed to provide an effect estimate and measure of precision from any analysis. This brings into question the validity and interpretability of the results and the conclusions drawn. The recommended approach for analysing QoL over time is the use of longitudinal analysis methods that account for the correlation of repeated measures such as joint longitudinal survival models, linear mixed effects models or generalised estimating equations, with the choice of model based on the estimand of interest, distribution of the outcome, and missing data mechanism. Models should adjust for baseline QoL scores and consider the impact of missing data due to disease progression/death.

## Supplementary Information

Supplementary Material 1.

Supplementary Material 2.

Supplementary Material 3.

Supplementary Material 4.

## Data Availability

The datasets used and/or analysed during the current study are available from the corresponding author on reasonable request.

## Abbreviations

ANOVA: Analysis of variance
CI: Confidence interval
CONSORT: Consolidated Standards of Reporting Trials
EORTC: European Organisation for Research and Treatment of Cancer
GEE: Generalised estimating equation
GLM: Generalised linear model
IQR: Interquartile range
MANOVA: Multivariate analysis of variance
MAR: Missing at random
MCAR: Missing completely at random
MCID: Minimal clinically important difference
PMM: Pattern mixture model
PRISMA: Preferred Reporting Items for Systematic Reviews and Meta-Analyses
QLQ-C30: Quality of Life Questionnaire Core 30
QLQ-LC13: Quality of Life Questionnaire Lung Cancer 13
QoL: Quality of life
RCT: Randomised controlled trial
SE: Standard error
STROBE: Strengthening the reporting of observational studies in epidemiology
TTE: Time to event
