# Supplementary material for: A scoping review of statistical methods used to report EORTC QLQ-C30 quality of life scores measured longitudinally
Source: BMC Med Res Methodol. 2025 Aug 2;25:188. doi: 10.1186/s12874-025-02622-1 (PMC12318403; doi:10.1186/s12874-025-02622-1)
Supplement: Supplementary file 2 — Supplementary Material 2. [file 12874_2025_2622_MOESM2_ESM.docx]

**Full Embase search strategy**

1. Randomized Controlled Trials as Topic/
2. random allocation/
3. double-blind method/
4. single-blind method/
5. random*.tw.
6. ((singl* or doubl* or trebl* or tripl*) adj (blind* or mask*)).tw.
7. (crossover* or cross-over).tw.
8. ((random* or control*) adj5 trial*).tw.
9. cohort studies/
10. cohort.tw.
11. or/1-10
12. cross-sectional study.tw. or cross-sectional study/ or (cross-sectional adj2 study).tw.
13. clinical protocol/ or protocol.ti.
14. statistical analysis plan.ti. or statistical analysis plan/ or economic evaluation/ or economic evaluation.kw. or systematic review/ or corrigendum.ti.
15. 12 or 13 or 14
16. 11 not 15
17. (EORTC Core-30 or EORTC Lung cancer-13 or Questionnaire-Core 30 or Questionnaire-Lung cancer 13 or QLQ-C30 or QLQ-LC13 or QLQ C30 or QLQ LC13 or QLQC30 or QLQLC13 or EORTC-QLQ-C30 or EORTC-QLQ-LC13 or EORTC-C30 or EORTC-LC13).af.
18. 16 and 17
19. (editorial or review or letter or conference abstract or note).pt.
20. 18 not 19
21. limit 20 to english language
22. limit 23 to yr=”2021 -2022”
