# Supplementary material for: A scoping review of statistical methods used to report EORTC QLQ-C30 quality of life scores measured longitudinally
Source: BMC Med Res Methodol. 2025 Aug 2;25:188. doi: 10.1186/s12874-025-02622-1 (PMC12318403; doi:10.1186/s12874-025-02622-1)
Supplement: Supplementary file 3 — Supplementary Material 3. [file 12874_2025_2622_MOESM3_ESM.pdf]

Study identifier

Record ID

Initials of person completing extraction

ORN

Title

Study details

Record ID

____

Study details

Study design

- ☐ Parallel group RCT
- ☐ Crossover RCT
- ☐ Factorial RCT
- ☐ Cluster randomised
- ☐ Cohort
- ☐ Data from multiple RCTs
- ☐ Data from multiple cohorts
- ☐ Pooled cohort from RCT
- ☐ Pilot/feasibility
- ☐ Other

If other design, specify

____

Which of the following questionnaires were used in the study

- ◯ QLQ-C30
- ◯ QLQ-LC13
- ◯ QLQ-C30 and QLQ-LC13

Was the QLQ-C30/QLQ-LC13 questionnaire completed at baseline

- ◯ Yes
- ◯ No

Frequency of follow up (excluding baseline)

____

Was any QLQ-C30 or QLQ-LC13 score the primary outcome

- ◯ Yes
- ◯ No
- ◯ Primary outcome not specified

If yes, what score was the primary outcome

____

Were the assumptions of the sample size calculation reported

- ◯ Yes
- ◯ No

If yes, what was the effect size used in the calculation e.g. 10 point difference in score

____

Was the same type of effect size presented from the primary outcome analysis?
e.g. mean difference used in sample size calculation and mean difference presented as primary outcome result

◯ Yes
◯ No

Effect size reported for primary outcome

____

Was a minimal clinically important difference (MCID) defined?

◯ Yes
◯ No

If yes, what was the MCID

____

Sample size

____

Number of patients included in analyses (answer unclear if so)

____

Analysis details

Record ID

____

Analysis details

Which scores derived from the questionnaires were formally analysed?

◯ All scores analysed (including or excluding financial difficulties)
◯ Only summary scores analysed (e.g. overall summary score, overall functioning score, overall symptom score)
◯ All scores analysed as well as a summary score
◯ A subset of scores analysed as well as a summary score
◯ Only a subset of scores analysed

Was any method used to account for missing questionnaires/scores (not missing data within a score)?

◯ Yes
◯ No - stated assumed missing at random (MAR)/missing completely at random (MCAR)
◯ No - and does not mention assuming MAR/MCAR

If yes, what method was used e.g. multiple imputation

____

Was any method used to account for missing data due to death?

◯ Yes
◯ No

If yes, what method was used?

____

Was more than one method used to analyse the QLQ-C30/QLQ-LC13 scores?

◯ Yes
◯ No

Model type 1 details

Model type used (method #1)

◯ Linear mixed effects model/repeated measures regression/mixed effects regression
◯ T-test
◯ Mann-Whitney U/Wilcoxon rank-sum test
◯ Wilcoxon signed-rank test
◯ Repeated measures ANOVA/MANCOVA
◯ ANOVA/ANCOVA
◯ Joint longitudinal survival model
◯ Ordinal logistic regression
◯ Logistic regression
◯ Linear regression
◯ Mixed-effects mixed-distribution models e.g. two-part models
◯ Time to event analysis e.g. time to deterioration
◯ Constrained longitudinal data analysis model
◯ Other

If other model used, specify

____

If time to event analysis, how was the event defined

____

If time to event analysis, what methods were used

☐ Cox proportional hazards model
☐ Kaplan-Meier methods
☐ Log-rank test
☐ Other model

If other, specify

____

If mixed-effect mixed-distribution model, what model was used?

____

For this model type, were scores from all time points analysed/included or a select few e.g. only last time point analysed

◯ All time points analysed/included
◯ Some time points not analysed

If some time points were not analysed or included in any models, provide details e.g. excluded middle time point

____

For this model type, what form of the score was modelled

◯ Raw score
◯ Change from baseline
◯ Both raw score and change from baseline modelled separately
◯ Mean/average of all scores over time
◯ Proportion achieving MID
◯ Other

If other, specify

____

For this model type, were the time points that were analysed included in one model or separate models

◯ One model for all time points analysed
◯ Separate models for each time point analysed
◯ Only one time point/summary score analysed
◯ Unclear

If all time points were included in one model, was time treated as categorical or continuous

◯ Categorical
◯ Continuous
◯ Unclear

Were any additional covariates included in the model other than group (if comparing by group)?

◯ Yes
◯ No
◯ Unclear

Were any additional covariates included in the model other than group (if comparing by group) and time?

◯ Yes
◯ No
◯ Unclear

If yes, specify covariates

____

For this first model type, were effect sizes & 95% CIs/SEs presented

◯ Yes both effect size and 95% CI/SE
◯ Effect size only
◯ Neither

For this first model type, were p-values presented

◯ Yes - only for main effect
◯ Yes - for main effect and time*treatment interaction
◯ Yes - only for time*treatment interaction
◯ Yes - only for change in score over time (within group or overall - not comparing by group)
◯ Yes - for main effect and change in score over time (within group or overall - not comparing by group)
◯ No p-values presented but indicated (e.g.*) where they were significant or below a threshold
◯ No p-values presented or indicated

Model type 2 details

Model type used (method #2)

◯ Linear mixed effects model/repeated measures regression/mixed effects regression
◯ T-test
◯ Mann-Whitney U/Wilcoxon rank-sum test
◯ Wilcoxon signed-rank test
◯ Repeated measures ANOVA/MANCOVA
◯ ANOVA/ANCOVA
◯ Joint longitudinal survival model
◯ Ordinal logistic regression
◯ Logistic regression
◯ Linear regression
◯ Mixed-effects mixed-distribution models e.g. two-part models
◯ Time to event analysis e.g. time to deterioration
◯ Constrained longitudinal data analysis model
◯ Other

If other model used, specify

____

If time to event analysis, how was the event defined

____

If time to event analysis, what methods were used

☐ Cox proportional hazards model
☐ Kaplan-Meier methods
☐ Log-rank test
☐ Other model

If other, specify

____

If mixed-effect mixed-distribution model, what model was used?

____

For this model type, were scores from all time points analysed/included or a select few e.g. only last time point analysed

◯ All time points analysed/included
◯ Some time points not analysed

If some time points were not analysed or included in any models, provide details e.g. excluded middle time point

____

For this model type, what form of the score was modelled

◯ Raw score
◯ Change from baseline
◯ Both raw score and change from baseline modelled separately
◯ Mean/average of all scores over time
◯ Proportion achieving MID
◯ Other

If other, specify

____

For this model type, were the time points that were analysed included in one model or separate models

◯ One model for all time points analysed
◯ Separate models for each time point analysed
◯ Only one time point/summary score analysed
◯ Unclear

If all time points were included in one model, was time treated as categorical or continuous

◯ Categorical
◯ Continuous
◯ Unclear

Were any additional covariates included in the model other than group (if comparing by group)?

◯ Yes
◯ No
◯ Unclear

Were any additional covariates included in the model other than group (if comparing by group) and time?

◯ Yes
◯ No
◯ Unclear

If yes, specify covariates

____

For this model type, were effect sizes & 95% CIs/SEs presented

◯ Yes both effect size and 95% CI/SE
◯ Effect size only
◯ Neither

For this model type, were p-values presented

◯ Yes - only for main effect
◯ Yes - for main effect and time*treatment interaction
◯ Yes - only for time*treatment interaction
◯ Yes - only for change in score over time (within group or overall - not comparing by group)
◯ Yes - for main effect and change in score over time (within group or overall - not comparing by group)
◯ No p-values presented but indicated (e.g.*) where they were significant or below a threshold
◯ No p-values presented or indicated

Was a third model type used to analyse the QLQ-C30/QLQ-LC13 scores

◯ Yes
◯ No

Model type 3 details

Model type used (method #3)

◯ Linear mixed effects model/repeated measures regression/mixed effects regression
◯ T-test
◯ Mann-Whitney U/Wilcoxon rank-sum test
◯ Wilcoxon signed-rank test
◯ Repeated measures ANOVA/MANCOVA
◯ ANOVA/ANCOVA
◯ Joint longitudinal survival model
◯ Ordinal logistic regression
◯ Logistic regression
◯ Linear regression
◯ Mixed-effects mixed-distribution models e.g. two-part models
◯ Time to event analysis e.g. time to deterioration
◯ Constrained longitudinal data analysis model
◯ Other

If other model used, specify

____

If time to event analysis, how was the event defined

____

If time to event analysis, what methods were used

☐ Cox proportional hazards model
☐ Kaplan-Meier methods
☐ Log-rank test
☐ Other model

If other, specify

____

If mixed-effect mixed-distribution model, what model was used?

____

For this model type, were scores from all time points analysed/included or a select few e.g. only last time point analysed

◯ All time points analysed/included
◯ Some time points not analysed

If some time points were not analysed or included in any models, provide details e.g. excluded middle time point

____

For this model type, what form of the score was modelled

◯ Raw score
◯ Change from baseline
◯ Both raw score and change from baseline modelled separately
◯ Mean/average of all scores over time
◯ Proportion achieving MID
◯ Other

If other, specify

____

For this model type, were the time points that were analysed included in one model or separate models

◯ One model for all time points analysed
◯ Separate models for each time point analysed
◯ Only one time point/summary score analysed
◯ Unclear

If all time points were included in one model, was time treated as categorical or continuous

◯ Categorical
◯ Continuous
◯ Unclear

Were any additional covariates included in the model other than group (if comparing by group)?

◯ Yes
◯ No
◯ Unclear

Were any additional covariates included in the model other than group (if comparing by group) and time?

◯ Yes
◯ No
◯ Unclear

If yes, specify covariates

____

For this model type, were effect sizes & 95% CIs/SEs presented

◯ Yes both effect size and 95% CI/SE
◯ Effect size only
◯ Neither

For this model type, were p-values presented

◯ Yes - only for main effect
◯ Yes - for main effect and time*treatment interaction
◯ Yes - only for time*treatment interaction
◯ Yes - only for change in score over time (within group or overall - not comparing by group)
◯ Yes - for main effect and change in score over time (within group or overall - not comparing by group)
◯ No p-values presented but indicated (e.g.*) where they were significant or below a threshold
◯ No p-values presented or indicated

Was a fourth model type used to analyse the QLQ-C30/QLQ-LC13 scores

◯ Yes
◯ No

Model type 4 details

Model type used (method #4)

◯ Linear mixed effects model/repeated measures regression/mixed effects regression
◯ T-test
◯ Mann-Whitney U/Wilcoxon rank-sum test
◯ Wilcoxon signed-rank test
◯ Repeated measures ANOVA/MANCOVA
◯ ANOVA/ANCOVA
◯ Joint longitudinal survival model
◯ Ordinal logistic regression
◯ Logistic regression
◯ Linear regression
◯ Mixed-effects mixed-distribution models e.g. two-part models
◯ Time to event analysis e.g. time to deterioration
◯ Constrained longitudinal data analysis model
◯ Other

If other model used, specify

____

If time to event analysis, how was the event defined

____

If time to event analysis, what methods were used

☐ Cox proportional hazards model
☐ Kaplan-Meier methods
☐ Log-rank test
☐ Other model

If other, specify

____

If mixed-effect mixed-distribution model, what model was used?

____

For this model type, were scores from all time points analysed/included or a select few e.g. only last time point analysed

◯ All time points analysed/included
◯ Some time points not analysed

If some time points were not analysed or included in any models, provide details e.g. excluded middle time point

____

For this model type, what form of the score was modelled

◯ Raw score
◯ Change from baseline
◯ Both raw score and change from baseline modelled separately
◯ Mean/average of all scores over time
◯ Proportion achieving MID
◯ Other

If other, specify

____

For this model type, were the time points that were analysed included in one model or separate models

◯ One model for all time points analysed
◯ Separate models for each time point analysed
◯ Only one time point/summary score analysed
◯ Unclear

If all time points were included in one model, was time treated as categorical or continuous

◯ Categorical
◯ Continuous
◯ Unclear

Were any additional covariates included in the model other than group (if comparing by group)?

◯ Yes
◯ No
◯ Unclear

Were any additional covariates included in the model other than group (if comparing by group) and time?

◯ Yes
◯ No
◯ Unclear

If yes, specify covariates

____

For this model type, were effect sizes & 95% CIs/SEs presented

◯ Yes both effect size and 95% CI/SE
◯ Effect size only
◯ Neither

For this model type, were p-values presented

◯ Yes - only for main effect
◯ Yes - for main effect and time*treatment interaction
◯ Yes - only for time*treatment interaction
◯ Yes - only for change in score over time (within group or overall - not comparing by group)
◯ Yes - for main effect and change in score over time (within group or overall - not comparing by group)
◯ No p-values presented but indicated (e.g.*) where they were significant or below a threshold
◯ No p-values presented or indicated

Was a fifth model type used to analyse the QLQ-C30/QLQ-LC13 scores

◯ Yes
◯ No

Model type 5 details

Model type used (method #5)

◯ Linear mixed effects model/repeated measures regression/mixed effects regression
◯ T-test
◯ Mann-Whitney U/Wilcoxon rank-sum test
◯ Wilcoxon signed-rank test
◯ Repeated measures ANOVA/MANCOVA
◯ ANOVA/ANCOVA
◯ Joint longitudinal survival model
◯ Ordinal logistic regression
◯ Logistic regression
◯ Linear regression
◯ Mixed-effects mixed-distribution models e.g. two-part models
◯ Time to event analysis e.g. time to deterioration
◯ Constrained longitudinal data analysis model
◯ Other

If other model used, specify

____

If time to event analysis, how was the event defined

____

If time to event analysis, what methods were used

☐ Cox proportional hazards model
☐ Kaplan-Meier methods
☐ Log-rank test
☐ Other model

If other, specify

____

If mixed-effect mixed-distribution model, what model was used?

____

For this model type, were scores from all time points analysed/included or a select few e.g. only last time point analysed

◯ All time points analysed/included
◯ Some time points not analysed

If some time points were not analysed or included in any models, provide details e.g. excluded middle time point

____

For this model type, what form of the score was modelled

◯ Raw score
◯ Change from baseline
◯ Both raw score and change from baseline modelled separately
◯ Mean/average of all scores over time
◯ Proportion achieving MID
◯ Other

If other, specify

____

For this model type, were the time points that were analysed included in one model or separate models

◯ One model for all time points analysed
◯ Separate models for each time point analysed
◯ Only one time point/summary score analysed
◯ Unclear

If all time points were included in one model, was time treated as categorical or continuous

◯ Categorical
◯ Continuous
◯ Unclear

Were any additional covariates included in the model other than group (if comparing by group)?

◯ Yes
◯ No
◯ Unclear

Were any additional covariates included in the model other than group (if comparing by group) and time?

◯ Yes
◯ No
◯ Unclear

If yes, specify covariates

____

For this model type, were effect sizes & 95% CIs/SEs presented

◯ Yes both effect size and 95% CI/SE
◯ Effect size only
◯ Neither

For this model type, were p-values presented

◯ Yes - only for main effect
◯ Yes - for main effect and time*treatment interaction
◯ Yes - only for time*treatment interaction
◯ Yes - only for change in score over time (within group or overall - not comparing by group)
◯ Yes - for main effect and change in score over time (within group or overall - not comparing by group)
◯ No p-values presented but indicated (e.g.*) where they were significant or below a threshold
◯ No p-values presented or indicated

Comments
