# Supplementary material for: A scoping review of statistical methods used to report EORTC QLQ-C30 quality of life scores measured longitudinally
Source: BMC Med Res Methodol. 2025 Aug 2;25:188. doi: 10.1186/s12874-025-02622-1 (PMC12318403; doi:10.1186/s12874-025-02622-1)
Supplement: Supplementary file 4 — Supplementary Material 4. [file 12874_2025_2622_MOESM4_ESM.pdf]

References of papers included in scoping review

1. Stavrinou M, Tsitsi T, Astras G, Paikousis L, Charalambous A. A randomised controlled feasibility trial to evaluate Lawsonia inermis (henna)'s effect on palmar-plantar erythrodysesthesia induced by capecitabine or pegylated liposomal doxorubicin. European Journal of Oncology Nursing. 2021;51:101908.
2. Clemons M, Ong M, Stober C, Ernst S, Booth C, Canil C, et al. A randomised trial of 4- versus 12-weekly administration of bone-targeted agents in patients with bone metastases from breast or castration-resistant prostate cancer. Eur J Cancer. 2021;142:132-40.
3. Xie H, Chen X, Xu L, Zhang R, Kang X, Wei X, et al. A randomized controlled trial of oral nutritional supplementation versus standard diet following McKeown minimally invasive esophagectomy in patients with esophageal malignancy: a pilot study. Annals of Translational Medicine. 2021;9(22):1674.
4. Klinghammer K, Fayette J, Kawecki A, Dietz A, Schafhausen P, Folprecht G, et al. A randomized phase II study comparing the efficacy and safety of the glyco-optimized anti-EGFR antibody tomuzotuximab against cetuximab in patients with recurrent and/or metastatic squamous cell cancer of the head and neck – the RESGEX study. ESMO Open. 2021;6(5):100242.
5. Qaderi SM, Swartjes H, Vromen H, Bremers AJA, Custers JAE, de Wilt JHW. Acceptability, quality of life and cost overview of a remote follow-up plan for patients with colorectal cancer. European Journal of Surgical Oncology. 2021;47(7):1637-44.
6. Tutt ANJ, Garber JE, Kaufman B, Viale G, Fumagalli D, Rastogi P, et al. Adjuvant Olaparib for Patients with BRCA1- or BRCA2-Mutated Breast Cancer. N Engl J Med. 2021;384(25):2394-405.
7. Bottomley A, Coens C, Mierzynska J, Blank CU, Mandalà M, Long GV, et al. Adjuvant pembrolizumab versus placebo in resected stage III melanoma (EORTC 1325-MG/KEYNOTE-054): health-related quality-of-life results from a double-blind, randomised, controlled, phase 3 trial. The Lancet Oncology. 2021;22(5):655-64.
8. Chi Y, Shu Y, Ba Y, Bai Y, Qin B, Wang X, et al. Anlotinib Monotherapy for Refractory Metastatic Colorectal Cancer: A Double-Blinded, Placebo-Controlled, Randomized Phase III Trial (ALTER0703). Oncologist. 2021;26(10):e1693-e703.
9. de Vries J, Bras L, Sidorenkov G, Festen S, Steenbakkers RJHM, Langendijk JA, et al. Association of Deficits Identified by Geriatric Assessment With Deterioration of Health-Related Quality of Life in Patients Treated for Head and Neck Cancer. JAMA Otolaryngology–Head & Neck Surgery. 2021;147(12):1089-99.
10. Battisti NML, Reed MWR, Herbert E, Morgan JL, Collins KA, Ward SE, et al. Bridging the Age Gap in breast cancer: Impact of chemotherapy on quality of life in older women with early breast cancer. Eur J Cancer. 2021;144:269-80.
11. Van Parijs H, Vinh-Hung V, Fontaine C, Storme G, Verschraegen C, Nguyen DM, et al. Cardiopulmonary-related patient-reported outcomes in a randomized clinical trial of radiation therapy for breast cancer. BMC Cancer. 2021;21(1):1177.
12. Pivodic L, De Burghgraeve T, Twisk J, van den Akker M, Buntinx F, Van den Block L. Changes in social, psychological and physical well-being in the last 5 years of life of older people with cancer: a longitudinal study. Age Ageing. 2021;50(5):1829-33.
13. Pérez-García JM, Gebhart G, Ruiz Borrego M, Stradella A, Bermejo B, Schmid P, et al. Chemotherapy de-escalation using an 18F-FDG-PET-based pathological response-adapted strategy in patients with HER2-positive early breast cancer (PHERGain): a multicentre, randomised, open-label, non-comparative, phase 2 trial. The Lancet Oncology. 2021;22(6):858-71.
14. Burbridge B, Lim H, Dwernychuk L, Le H, Asif T, Sami A, et al. Comparison of the Quality of Life of Patients with Breast or Colon Cancer with an Arm Vein Port (TIVAD) Versus a Peripherally Inserted Central Catheter (PICC). Curr Oncol. 2021;28(2):1495-506.
15. Poon DMC, Kam MKM, Johnson D, Mo F, Tong M, Chan ATC. Durability of the parotid-sparing effect of intensity-modulated radiotherapy (IMRT) in early stage nasopharyngeal carcinoma: A 15-year follow-up of a randomized prospective study of IMRT versus two-dimensional radiotherapy. Head & Neck. 2021;43(6):1711-20.
16. Wu X, Liu Y, Zhu D, Wang F, Ji J, Yan H. Early prevention of complex decongestive therapy and rehabilitation exercise for prevention of lower extremity lymphedema after operation of gynecologic cancer. Asian Journal of Surgery. 2021;44(1):111-5.
17. Nottelmann L, Groenvold M, Vejlgaard TB, Petersen MA, Jensen LH. Early, integrated palliative rehabilitation improves quality of life of patients with newly diagnosed advanced cancer: The Pal-Rehab randomized controlled trial. Palliative Medicine. 2021;35(7):1344-55.

18. Khantwal G, Sharma SK, Rani R, Agarwal SP. Effect of Postsurgical Nurse-led Follow-ups on Quality of Life in Head-and-Neck Cancer Patients: A Pilot Randomized Controlled Trial. Asia-Pacific Journal of Oncology Nursing. 2021;8(5):573-80.
19. Vos JAM, Duineveld LAM, Wieldraaijer T, Wind J, Busschers WB, Sert E, et al. Effect of general practitioner-led versus surgeon-led colon cancer survivorship care, with or without eHealth support, on quality of life (I CARE): an interim analysis of 1-year results of a randomised, controlled trial. The Lancet Oncology. 2021;22(8):1175-87.
20. Lohmander F, Lagergren J, Johansson H, Roy PG, Brandberg Y, Frisell J. Effect of Immediate Implant-Based Breast Reconstruction After Mastectomy With and Without Acellular Dermal Matrix Among Women With Breast Cancer: A Randomized Clinical Trial. JAMA Network Open. 2021;4(10):e2127806-e.
21. Patrick DL, Powers A, Jun MP, Kim Y, Garcia J, Dehner C, et al. Effect of lisocabtagene maraleucel on HRQoL and symptom severity in relapsed/refractory large B-cell lymphoma. Blood Advances. 2021;5(8):2245-55.
22. Koet LL, Kraima A, Derksen I, Lamme B, Belt EJT, van Rosmalen J, et al. Effectiveness of preoperative group education for patients with colorectal cancer: managing expectations. Supportive Care in Cancer. 2021;29(9):5263-71.
23. Takano T, Matsuda A, Ishizuka N, Ozaki Y, Suyama K, Tanabe Y, et al. Effectiveness of self-help workbook intervention on quality of life in cancer patients receiving chemotherapy: results of a randomized controlled trial. BMC Cancer. 2021;21(1):588.
24. Chen Y-H, Huang C-Y, Liang W-A, Lin C-R, Chao Y-H. Effects of Conscious Control of Scapular Orientation in Oral Cancer Survivors With Scapular Dyskinesis: A Randomized Controlled Trial. Integrative Cancer Therapies. 2021;20:15347354211040827.
25. Han X, Lu Y, Fang Q, Fang P, Wong GTC, Liu X. Effects of Epidural Anesthesia on Quality of Life in Elderly Patients Undergoing Esophagectomy. Seminars in Thoracic and Cardiovascular Surgery. 2021;33(1):276-85.
26. Amabile MI, De Luca A, Tripodi D, D'Alberti E, Melcarne R, Imbimbo G, et al. Effects of Inositol Hexaphosphate and Myo-Inositol Administration in Breast Cancer Patients during Adjuvant Chemotherapy. J Pers Med. 2021;11(8).
27. Morielli AR, Boulé NG, Usmani N, Tankel K, Joseph K, Severin D, et al. Effects of exercise during and after neoadjuvant chemoradiation on symptom burden and quality of life in rectal cancer patients: a phase II randomized controlled trial. Journal of Cancer Survivorship. 2023;17(4):1171-83.
28. Yoon BJ, Oh H-K, Lee J, Cho JR, Kim MJ, Kim D-W, et al. Effects of probiotics on bowel function restoration following ileostomy closure in rectal cancer patients: a randomized controlled trial. Colorectal Disease. 2021;23(4):901-10.
29. Yang JC-H, Mok TSK, Lu S, Nakagawa K, Yamamoto N, Shi Y-K, et al. Efficacy and Safety of S-1 Compared With Docetaxel in Elderly Patients With Advanced NSCLC Previously Treated With Platinum-Based Chemotherapy: A Subgroup Analysis of the EAST-LC Trial. JTO Clinical and Research Reports. 2021;2(3):100142.
30. Ram Z, Kim C-Y, Hottinger AF, Idbaih A, Nicholas G, Zhu J-J. Efficacy and Safety of Tumor Treating Fields (TTFields) in Elderly Patients with Newly Diagnosed Glioblastoma: Subgroup Analysis of the Phase 3 EF-14 Clinical Trial. Frontiers in Oncology. 2021;Volume 11 - 2021.
31. Gresham G, Placencio-Hickok VR, Lauzon M, Nguyen T, Kim H, Mehta S, et al. Feasibility and efficacy of enteral tube feeding on weight stability, lean body mass, and patient-reported outcomes in pancreatic cancer cachexia. Journal of Cachexia, Sarcopenia and Muscle. 2021;12(6):1959-68.
32. Koole SN, Kieffer JM, K.Sikorska, Schagen van Leeuwen JH, Schreuder HWR, Hermans RH, et al. Health-related quality of life after interval cytoreductive surgery with or without hyperthermic intraperitoneal chemotherapy (HIPEC) in patients with stage III ovarian cancer. European Journal of Surgical Oncology. 2021;47(1):101-7.
33. Klevebro F, Kauppila JH, Markar S, Johar A, Lagergren P. Health-related quality of life following total minimally invasive, hybrid minimally invasive or open oesophagectomy: a population-based cohort study. British Journal of Surgery. 2020;108(6):702-8.
34. Maurer T, Thöne K, Obi N, Jung AY, Behrens S, Becher H, et al. Health-Related Quality of Life in a Cohort of Breast Cancer Survivors over More Than 10 Years Post-Diagnosis and in Comparison to a Control Cohort. Cancers (Basel). 2021;13(8).
35. Van Cutsem E, Amonkar M, Fuchs CS, Alsina M, Özgüroğlu M, Bang YJ, et al. Health-related quality of life in advanced gastric/gastroesophageal junction cancer with second-line pembrolizumab in KEYNOTE-061. Gastric Cancer. 2021;24(6):1330-40.
36. Andre T, Amonkar M, Norquist JM, Shiu K-K, Kim TW, Jensen BV, et al. Health-related quality of life in patients with microsatellite instability-high or mismatch repair deficient metastatic colorectal cancer treated

with first-line pembrolizumab versus chemotherapy (KEYNOTE-177): an open-label, randomised, phase 3 trial. The Lancet Oncology. 2021;22(5):665-77.
37. Knop S, Mateos M-V, Dimopoulos MA, Suzuki K, Jakubowiak A, Doyen C, et al. Health-related quality of life in patients with newly diagnosed multiple myeloma ineligible for stem cell transplantation: results from the randomized phase III ALCYONE trial. BMC Cancer. 2021;21(1):659.
38. Plesner T, Dimopoulos MA, Oriol A, San-Miguel J, Bahlis NJ, Rabin N, et al. Health-related quality of life in patients with relapsed or refractory multiple myeloma: treatment with daratumumab, lenalidomide, and dexamethasone in the phase 3 POLLUX trial. British Journal of Haematology. 2021;194(1):132-9.
39. Garcia Campelo MR, Lin HM, Zhu Y, Pérol M, Jahanzeb M, Popat S, et al. Health-related quality of life in the randomized phase III trial of brigatinib vs crizotinib in advanced ALK inhibitor–naive ALK + non–small cell lung cancer (ALTA-1L). Lung Cancer. 2021;155:68-77.
40. Perrot A, Facon T, Plesner T, Usmani SZ, Kumar S, Bahlis NJ, et al. Health-Related Quality of Life in Transplant-Ineligible Patients With Newly Diagnosed Multiple Myeloma: Findings From the Phase III MAIA Trial. Journal of Clinical Oncology. 2021;39(3):227-37.
41. Hungria V, Beksac M, Weisel KC, Nooka AK, Masszi T, Spicka I, et al. Health-related quality of life maintained over time in patients with relapsed or refractory multiple myeloma treated with daratumumab in combination with bortezomib and dexamethasone: results from the phase III CASTOR trial. British Journal of Haematology. 2021;193(3):561-9.
42. Jacobs DHM, Charaghvandi RK, Horeweg N, Maduro JH, Speijer G, Roeloffzen EMA, et al. Health-related quality of life of early-stage breast cancer patients after different radiotherapy regimens. Breast Cancer Res Treat. 2021;189(2):387-98.
43. Al-Sawaf O, Gentile B, Devine J, Zhang C, Sail K, Tandon M, et al. Health-related quality of life with fixed-duration venetoclax-obinutuzumab for previously untreated chronic lymphocytic leukemia: Results from the randomized, phase 3 CLL14 trial. American Journal of Hematology. 2021;96(9):1112-9.
44. Kahan Z, Gil-Gil M, Ruiz-Borrego M, Carrasco E, Ciruelos E, Muñoz M, et al. Health-related quality of life with palbociclib plus endocrine therapy versus capecitabine in postmenopausal patients with hormone receptor–positive metastatic breast cancer: Patient-reported outcomes in the PEARL study. European Journal of Cancer. 2021;156:70-82.
45. Ryoo B-Y, Merle P, Kulkarni AS, Cheng A-L, Bouattour M, Lim HY, et al. Health-related quality-of-life impact of pembrolizumab versus best supportive care in previously systemically treated patients with advanced hepatocellular carcinoma: KEYNOTE-240. Cancer. 2021;127(6):865-74.
46. Clement PMJ, Dirven L, Eoli M, Sepulveda-Sanchez JM, Walenkamp AME, Frenel JS, et al. Impact of depatuxizumab mafodotin on health-related quality of life and neurological functioning in the phase II EORTC 1410/INTELLANCE 2 trial for EGFR-amplified recurrent glioblastoma. European Journal of Cancer. 2021;147:1-12.
47. van der Laan HP, Van den Bosch L, Schuit E, Steenbakkers RJHM, van der Schaaf A, Langendijk JA. Impact of radiation-induced toxicities on quality of life of patients treated for head and neck cancer. Radiotherapy and Oncology. 2021;160:47-53.
48. Rischin D, Khushalani NI, Schmults CD, Guminski A, Chang ALS, Lewis KD, et al. Integrated analysis of a phase 2 study of cemiplimab in advanced cutaneous squamous cell carcinoma: extended follow-up of outcomes and quality of life analysis. J Immunother Cancer. 2021;9(8).
49. Post CCB, de Boer SM, Powell ME, Mileshkin L, Katsaros D, Bessette P, et al. Long-Term Toxicity and Health-Related Quality of Life After Adjuvant Chemoradiation Therapy or Radiation Therapy Alone for High-Risk Endometrial Cancer in the Randomized PORTEC-3 Trial. International Journal of Radiation Oncology*Biology*Physics. 2021;109(4):975-86.
50. Volz Y, Eismann L, Pfitzinger P, Westhofen T, Ebner B, Jokisch J-F, et al. Long-term Health-related Quality of Life (HRQOL) After Radical Cystectomy and Urinary Diversion - A Propensity Score-matched Analysis. Clinical Genitourinary Cancer. 2022;20(4):e283-e90.
51. Hoffmann C, Rating P, Bechrakis N, Eckstein A, Sokolenko E, Jabbarli L, et al. Long-term follow-up and health-related quality of life among cancer survivors with stage IEA orbital-type lymphoma after external photon-beam radiotherapy: Results from a longitudinal study. Hematological Oncology. 2022;40(5):922-9.
52. Tran TXM, Jung S-Y, Lee E-G, Cho H, Cho J, Lee E, et al. Long-term trajectory of postoperative health-related quality of life in young breast cancer patients: a 15-year follow-up study. Journal of Cancer Survivorship. 2023;17(5):1416-26.
53. Thomas CM, Sklar MC, Su J, Xu W, De Almeida JR, Alibhai SMH, et al. Longitudinal Assessment of Frailty and Quality of Life in Patients Undergoing Head and Neck Surgery. The Laryngoscope. 2021;131(7):E2232-E42.

54. Querido NR, Kenkhuis M-F, van Roekel EH, Breukink SO, van Duijnhoven FJB, Janssen-Heijnen MLG, et al. Longitudinal Associations between Inflammatory Markers and Fatigue up to Two Years after Colorectal Cancer Treatment. Cancer Epidemiology, Biomarkers & Prevention. 2022;31(8):1638-49.
55. Kenkhuis MF, van Duijnhoven FJB, van Roekel EH, Breedveld-Peters JJL, Breukink SO, Janssen-Heijnen ML, et al. Longitudinal associations of fiber, vegetable, and fruit intake with quality of life and fatigue in colorectal cancer survivors up to 24 months posttreatment. Am J Clin Nutr. 2022;115(3):822-32.
56. Brusniak K, Feisst M, Sebesteny L, Hartkopf A, Graf J, Engler T, et al. Measuring the Time to Deterioration for Health-Related Quality of Life in Patients With Metastatic Breast Cancer Using a Web-Based Monitoring Application: Longitudinal Cohort Study. JMIR Cancer. 2021;7(4):e25776.
57. Lei Y, Ho SC, Kwok C, Cheng A, Cheung KL, Lee R, et al. Menopausal symptoms inversely associated with quality of life: findings from a 5-year longitudinal cohort in Chinese breast cancer survivors. Menopause. 2021;28(8).
58. Ko E-J, Kwag E-B, Park JH, Park S-J, Son J-W, Yoon S-H, et al. Multi-Center, Randomized, Double-Blind, Placebo-Controlled, Exploratory Study to Evaluate the Efficacy and Safety of HAD-B1 for Dose-Finding in EGFR Mutation Positive and Locally Advanced or Metastatic NSCLC Subjects Who Need Afatinib Therapy. Integrative Cancer Therapies. 2021;20:15347354211037917.
59. Patursson P, Møller G, Muhic A, Andersen JR. N-3 fatty acid EPA supplementation in cancer patients receiving abdominal radiotherapy - A randomised controlled trial. Clinical Nutrition ESPEN. 2021;43:130-6.
60. Moy B, Oliveira M, Saura C, Gradishar W, Kim S-B, Brufsky A, et al. Neratinib + capecitabine sustains health-related quality of life in patients with HER2-positive metastatic breast cancer and ≥ 2 prior HER2-directed regimens. Breast Cancer Research and Treatment. 2021;188(2):449-58.
61. Wefel JS, Armstrong TS, Pugh SL, Gilbert MR, Wendland MM, Brachman DG, et al. Neurocognitive, symptom, and health-related quality of life outcomes of a randomized trial of bevacizumab for newly diagnosed glioblastoma (NRG/RTOG 0825). Neuro Oncol. 2021;23(7):1125-38.
62. Schwartz CE, Stark RB, Borowiec K, Myren K-J. No impact of Asian ethnicity on EORTC QLQ-C30 scores: Group differences and differential item functioning in paroxysmal nocturnal hemoglobinuria. Health and Quality of Life Outcomes. 2021;19(1):228.
63. de Souza APS, da Silva LC, Fayh APT. Nutritional Intervention Contributes to the Improvement of Symptoms Related to Quality of Life in Breast Cancer Patients Undergoing Neoadjuvant Chemotherapy: A Randomized Clinical Trial. Nutrients. 2021;13(2):589.
64. Frangou E, Bertelli G, Love S, Mackean MJ, Glasspool RM, Fotopoulou C, et al. OVPSYCH2: A randomized controlled trial of psychological support versus standard of care following chemotherapy for ovarian cancer. Gynecologic Oncology. 2021;162(2):431-9.
65. Battisti NML, Hatton MQ, Reed MWR, Herbert E, Morgan JL, Bradburn M, et al. Observational cohort study in older women with early breast cancer: Use of radiation therapy and impact on health-related quality of life and mortality. Radiotherapy and Oncology. 2021;161:166-76.
66. Garon EB, Cho BC, Reinmuth N, Lee KH, Luft A, Ahn M-J, et al. Patient-Reported Outcomes with Durvalumab With or Without Tremelimumab Versus Standard Chemotherapy as First-Line Treatment of Metastatic Non–Small-Cell Lung Cancer (MYSTIC). Clinical Lung Cancer. 2021;22(4):301-12.e8.
67. Van den Bosch L, van der Laan HP, van der Schaaf A, Oosting SF, Halmos GB, Witjes MJH, et al. Patient-Reported Toxicity and Quality-of-Life Profiles in Patients With Head and Neck Cancer Treated With Definitive Radiation Therapy or Chemoradiation. International Journal of Radiation Oncology*Biology*Physics. 2021;111(2):456-67.
68. Boevé L, Hulshof MCCM, Verhagen PCMS, Twisk JWR, Witjes WPJ, de Vries P, et al. Patient-reported Quality of Life in Patients with Primary Metastatic Prostate Cancer Treated with Androgen Deprivation Therapy with and Without Concurrent Radiation Therapy to the Prostate in a Prospective Randomised Clinical Trial; Data from the HORRAD Trial. European Urology. 2021;79(2):188-97.
69. Bines J, Clark E, Barton C, Restuccia E, Procter M, Sonnenblick A, et al. Patient-reported function, health-related quality of life, and symptoms in APHINITY: pertuzumab plus trastuzumab and chemotherapy in HER2-positive early breast cancer. Br J Cancer. 2021;125(1):38-47.
70. Lordick F, Al-Batran SE, Ganguli A, Morlock R, Sahin U, Türeci Ö. Patient-reported outcomes from the phase II FAST trial of zolbetuximab plus EOX compared to EOX alone as first-line treatment of patients with metastatic CLDN18.2+ gastroesophageal adenocarcinoma. Gastric Cancer. 2021;24(3):721-30.
71. Ciruelos EM, Rugo HS, Mayer IA, Levy C, Forget F, Mingorance JID, et al. Patient-Reported Outcomes in Patients With <i>PIK3CA</i>-Mutated Hormone Receptor–Positive, Human Epidermal Growth Factor Receptor 2–Negative Advanced Breast Cancer From SOLAR-1. Journal of Clinical Oncology. 2021;39(18):2005-15.

72. Galle PR, Finn RS, Qin S, Ikeda M, Zhu AX, Kim T-Y, et al. Patient-reported outcomes with atezolizumab plus bevacizumab versus sorafenib in patients with unresectable hepatocellular carcinoma (IMbrave150): an open-label, randomised, phase 3 trial. The Lancet Oncology. 2021;22(7):991-1001.
73. Sharma A, Singh M, Chauhan R, Malik PS, Khurana S, Mathur S, et al. Pazopanib based oral metronomic therapy for platinum resistant/refractory epithelial ovarian cancer: A phase II, open label, randomized, controlled trial. Gynecologic Oncology. 2021;162(2):382-8.
74. Absolom K, Warrington L, Hudson E, Hewison J, Morris C, Holch P, et al. Phase III Randomized Controlled Trial of eRAPID: eHealth Intervention During Chemotherapy. Journal of Clinical Oncology. 2021;39(7):734-47.
75. Karlsen J, Torgrim T, Piotr S, Øyvind S, S. SJ, Steinar L, et al. Pneumonitis and fibrosis after breast cancer radiotherapy: occurrence and treatment-related predictors. Acta Oncologica. 2021;60(12):1651-8.
76. Lindberg Å, Eskelund CW, Albertsson-Lindblad A, Kolstad A, Laurell A, Räty R, et al. Pre-treatment health-related quality of life parameters have prognostic impact in patients >65 years with newly diagnosed mantle cell lymphoma: The Nordic Lymphoma Group MCL4 (LENA-BERIT) experience. Hematological Oncology. 2022;40(1):23-31.
77. Müller J, Weiler M, Schneeweiss A, Haag GM, Steindorf K, Wick W, et al. Preventive effect of sensorimotor exercise and resistance training on chemotherapy-induced peripheral neuropathy: a randomised-controlled trial. British Journal of Cancer. 2021;125(7):955-65.
78. Berezowska A, Passchier E, Bleiker E. Professional patient navigation in a hospital setting: a randomized controlled trial. Supportive Care in Cancer. 2021;29(4):2111-23.
79. Maldonado F, Gonzalez-Ling A, Oñate-Ocaña LF, Cabrera-Miranda LA, Zatarain-Barrón ZL, Turcott JG, et al. Prophylactic Cranial Irradiation in Patients With High-Risk Metastatic Non-Small Cell Lung Cancer: Quality of Life and Neurocognitive Analysis of a Randomized Phase II Study. International Journal of Radiation Oncology*Biology*Physics. 2021;111(1):81-92.
80. Song YC, Sun GY, Fang H, Tang Y, Song YW, Hu C, et al. Quality of Life After Partial or Whole-Breast Irradiation in Breast-Conserving Therapy for Low-Risk Breast Cancer: 1-Year Results of a Phase 2 Randomized Controlled Trial. Front Oncol. 2021;11:738318.
81. Forster T, Hommertgen A, Häfner MF, Arians N, König L, Harrabi SB, et al. Quality of life after simultaneously integrated boost with intensity-modulated versus conventional radiotherapy with sequential boost for adjuvant treatment of breast cancer: 2-year results of the multicenter randomized IMRT-MC2 trial. Radiotherapy and Oncology. 2021;163:165-76.
82. Yamaguchi K, Shimada Y, Hironaka S, Sugimoto N, Komatsu Y, Nishina T, et al. Quality of Life Associated with Ramucirumab Treatment in Patients with Advanced Gastric Cancer in Japan: Exploratory Analysis from the Phase III RAINBOW Trial. Clin Drug Investig. 2021;41(1):53-64.
83. Wong P, Lambert L, Thanomsack P, Coulombe G, Lambert C, Charpentier A-M, et al. Quality of Life: A Prospective Randomized Trial of Palliative Volumetric Arc Therapy Versus 3-Dimensional Conventional Radiation Therapy. International Journal of Radiation Oncology*Biology*Physics. 2021;109(5):1431-9.
84. Kang S-B, Cho JR, Jeong S-Y, Oh JH, Ahn S, Choi S, et al. Quality of life after sphincter preservation surgery or abdominoperineal resection for low rectal cancer (ASPIRE): A long-term prospective, multicentre, cohort study. The Lancet Regional Health - Western Pacific. 2021;6:100087.
85. Lugtenberg RT, de Groot S, Kaptein AA, Fischer MJ, Kranenbarg EM, Carpentier MD, et al. Quality of life and illness perceptions in patients with breast cancer using a fasting mimicking diet as an adjunct to neoadjuvant chemotherapy in the phase 2 DIRECT (BOOG 2013-14) trial. Breast Cancer Res Treat. 2021;185(3):741-58.
86. Kang BM, Lee YS, Kim JH, Kim HJ, Lee SC, Kim CW, et al. Quality of life and patient satisfaction after single- and multiport laparoscopic surgery in colon cancer: a multicentre randomised controlled trial (SIMPLE Trial). Surgical Endoscopy. 2021;35(11):6278-90.
87. Liao K-C, Chuang H-C, Chien C-Y, Lin Y-T, Tsai M-H, Su Y-Y, et al. Quality of Life as a Mediator between Cancer Stage and Long-Term Mortality in Nasopharyngeal Cancer Patients Treated with Intensity-Modulated Radiotherapy. Cancers. 2021;13(20):5063.
88. Menon N, Patil V, Noronha V, Joshi A, Bhattacharjee A, Satam BJ, et al. Quality of life in patients with locally advanced head and neck cancer treated with concurrent chemoradiation with cisplatin and nimotuzumab versus cisplatin alone – Additional data from a phase 3 trial. Oral Oncology. 2021;122:105517.
89. Kosmala R, Fokas E, Flentje M, Sauer R, Liersch T, Graeven U, et al. Quality of life in rectal cancer patients with or without oxaliplatin in the randomised CAO/ARO/AIO-04 phase 3 trial. European Journal of Cancer. 2021;144:281-90.

90. Liposits G, Eshøj HR, Möller S, Winther SB, Skuladottir H, Ryg J, et al. Quality of Life in Vulnerable Older Patients with Metastatic Colorectal Cancer Receiving Palliative Chemotherapy-The Randomized NORDIC9-Study. Cancers (Basel). 2021;13(11).
91. Van Cutsem E, Valderrama A, Bang YJ, Fuchs CS, Shitara K, Janjigian YY, et al. Quality of life with first-line pembrolizumab for PD-L1–positive advanced gastric/gastroesophageal junction adenocarcinoma: results from the randomised phase III KEYNOTE-062 study. ESMO Open. 2021;6(4):100189.
92. Bach SP, Gilbert A, Brock K, Korsgen S, Geh I, Hill J, et al. Radical surgery versus organ preservation via short-course radiotherapy followed by transanal endoscopic microsurgery for early-stage rectal cancer (TREC): a randomised, open-label feasibility study. The Lancet Gastroenterology & Hepatology. 2021;6(2):92-105.
93. Deschuymer S, Nevens D, Duprez F, Daisne JF, Voordeckers M, De Neve W, et al. Randomized Clinical Trial on Reduction of Radiotherapy Dose to the Elective Neck in Head and Neck Squamous Cell Carcinoma: Results on the Quality of Life. Quality of Life Research. 2021;30(1):117-27.
94. Van Der Weijst L, Surmont V, Schrauwen W, Lievens Y. Real Life Data on Patient-Reported Outcomes and Neuro-Cognitive Functioning of Lung Cancer Patients: The PRO-Long Study. Front Oncol. 2021;11:685605.
95. Hathiramani S, Pettengell R, Moir H, Younis A. Relaxation versus exercise for improved quality of life in lymphoma survivors—a randomised controlled trial. Journal of Cancer Survivorship. 2021;15(3):470-80.
96. Zhang L, Li Y, Kou W, Xia Y, Yu X, Du X. Reminiscence therapy exhibits alleviation of anxiety and improvement of life quality in postoperative gastric cancer patients: A randomized, controlled study. Medicine. 2021;100(35):e26821.
97. Liu M, Li Y. Reminiscence therapy-based care program relieves anxiety, depression, and improves quality of life in post-operational non-small cell lung cancer patients. The Clinical Respiratory Journal. 2021;15(5):472-81.
98. Johnson ML, Zvirbule Z, Laktionov K, Helland A, Cho BC, Gutierrez V, et al. Rovalpituzumab Tesirine as a Maintenance Therapy After First-Line Platinum-Based Chemotherapy in Patients With Extensive-Stage–SCLC: Results From the Phase 3 MERU Study. Journal of Thoracic Oncology. 2021;16(9):1570-81.
99. Wang J, Ma X, Shang K, Wu S, Ma Y, Ma Z, et al. Safety and efficacy of spleen aminopeptide oral lyophilized powder for improving quality of life and immune response in patients with advanced breast cancer: a multicenter, randomized, double-blind, placebo-controlled clinical trial. Anti-Cancer Drugs. 2021;32(10):1067-75.
100. Volz Y, Eismann L, Pfitzinger PL, Jokisch J-F, Schulz G, Rodler S, et al. Salvage cystectomy and ileal conduit urinary diversion as a last-line option for benign diseases—perioperative safety and postoperative health-related quality of life. Neurourology and Urodynamics. 2021;40(5):1154-64.
101. Sahgal A, Myrehaug SD, Siva S, Masucci GL, Maralani PJ, Brundage M, et al. Stereotactic body radiotherapy versus conventional external beam radiotherapy in patients with painful spinal metastases: an open-label, multicentre, randomised, controlled, phase 2/3 trial. The Lancet Oncology. 2021;22(7):1023-33.
102. Tsai YT, Chen WC, Hsu CM, Tsai MS, Chang GH, Lee YC, et al. Survival-Weighted Health Profiles in Patients Treated for Advanced Oral Cavity Squamous Cell Carcinoma. Front Oncol. 2021;11:754412.
103. Nielsen AWM, Marie L, Melgaard NH, Jan A, Birgitte VO, Horsholt KM, et al. Symptom trajectories in breast cancer survivors: growth mixture analysis of patient-reported pain, fatigue, insomnia, breast and arm symptoms. Acta Oncologica. 2021;60(12):1659-67.
104. Pujalte Martin M, Borchiellini D, Thamphya B, Guillot A, Paoli J-B, Besson D, et al. TAXOMET: A French Prospective Multicentric Randomized Phase II Study of Docetaxel Plus Metformin Versus Docetaxel Plus Placebo in Metastatic Castration-Resistant Prostate Cancer. Clinical Genitourinary Cancer. 2021;19(6):501-9.
105. Molassiotis A, Vu DV, Ching SSY. The Effectiveness of Qigong in Managing a Cluster of Symptoms (Breathlessness-Fatigue-Anxiety) in Patients with Lung Cancer: A Randomized Controlled Trial. Integr Cancer Ther. 2021;20:15347354211008253.
106. Kargo AS, Tine JP, Kristina L, Ingvar HNH, Bente L, Mette H, et al. The PROMova study comparing active and passive use of patient-reported outcome measures in ovarian cancer follow-up: effect on patient-perceived involvement, satisfaction with care, and usefulness. Acta Oncologica. 2021;60(4):434-43.
107. Bonhof CS, van de Poll-Franse LV, Wasowicz DK, Beerepoot LV, Vreugdenhil G, Mols F. The course of peripheral neuropathy and its association with health-related quality of life among colorectal cancer patients. Journal of Cancer Survivorship. 2021;15(2):190-200.
108. Zhou L, Sun H. The effect of reminiscence therapy-involved care on anxiety, depression, life quality and survival in colorectal cancer patients. Clinics and Research in Hepatology and Gastroenterology. 2021;45(3):101546.
109. Loughney L, West MA, Moyses H, Bates A, Kemp GJ, Hawkins L, et al. The effects of neoadjuvant chemoradiotherapy and an in-hospital exercise training programme on physical fitness and quality of life in

locally advanced rectal cancer patients: a randomised controlled trial (The EMPOWER Trial). Perioperative Medicine. 2021;10(1):23.
110. Youn SI, Son SY, Lee K, Won Y, Min S, Park YS, et al. Quality of life after laparoscopic sentinel node navigation surgery in early gastric cancer: a single-center cohort study. Gastric Cancer. 2021;24(3):744-51.
111. Batenburg MCT, Maarse W, van der Leij F, Baas IO, Boonstra O, Lansdorp N, et al. The impact of hyperbaric oxygen therapy on late radiation toxicity and quality of life in breast cancer patients. Breast Cancer Research and Treatment. 2021;189(2):425-33.
112. Paty J, Sandin R, Reisman A, Wu YL, Migliorino MR, Zhou X, et al. The patient's perspective on treatment with dacomitinib: patient-reported outcomes from the Phase III trial ARCHER 1050. Future Oncol. 2021;17(7):783-94.
113. Qaderi SM, van der Heijden JAG, Verhoeven RHA, de Wilt JHW, Custers JAE, Beets GL, et al. Trajectories of health-related quality of life and psychological distress in patients with colorectal cancer: A population-based study. European Journal of Cancer. 2021;158:144-55.
114. Tabernero J, Shitara K, Zaanan A, Doi T, Lorenzen S, Van Cutsem E, et al. Trifluridine/tipiracil versus placebo for third or later lines of treatment in metastatic gastric cancer: an exploratory subgroup analysis from the TAGS study. ESMO Open. 2021;6(4):100200.
115. Fransson P, Nilsson P, Gunnlaugsson A, Beckman L, Tavelin B, Norman D, et al. Ultra-hypofractionated versus conventionally fractionated radiotherapy for prostate cancer (HYPO-RT-PC): patient-reported quality-of-life outcomes of a randomised, controlled, non-inferiority, phase 3 trial. The Lancet Oncology. 2021;22(2):235-45.
116. Chien A, Yang C-C, Chang S-C, Jan Y-M, Yang C-H, Hsieh Y-L. Ultrasound Acupuncture for Oxaliplatin-induced Peripheral Neuropathy in Patients With Colorectal Cancer: A Pilot Study. PM&R. 2021;13(1):55-65.
117. Keum J, Chung MJ, Kim Y, Ko H, Sung MJ, Jo JH, et al. Usefulness of Smartphone Apps for Improving Nutritional Status of Pancreatic Cancer Patients: Randomized Controlled Trial. JMIR Mhealth Uhealth. 2021;9(8):e21088.
118. D'Andréa G, Bordenave L, Nguyen F, Tao Y, Paleri V, Temam S, et al. A prospective longitudinal study of quality of life in robotic-assisted salvage surgery for oropharyngeal cancer. European Journal of Surgical Oncology. 2022;48(6):1243-50.
119. Khattak MA, Luke JJ, Long GV, Ascierto PA, Rutkowski P, Schadendorf D, et al. Adjuvant pembrolizumab versus placebo in resected high-risk stage II melanoma: Health-related quality of life from the randomized phase 3 KEYNOTE-716 study. European Journal of Cancer. 2022;176:207-17.
120. Orive M, Anton-Ladislao A, Lázaro S, Gonzalez N, Bare M, Fernandez de Larrea N, et al. Anxiety, depression, health-related quality of life, and mortality among colorectal patients: 5-year follow-up. Support Care Cancer. 2022;30(10):7943-54.
121. Miyake M, Nishimura N, Ohnishi S, Oda Y, Miyamoto T, Shimizu T, et al. Association between urine 6-sulfatoxy-melatonin level and intravesical Bacillus Calmette-Guerin treatment–induced sleep quality deterioration in patients with non-muscle invasive bladder cancer. Supportive Care in Cancer. 2022;30(7):6145-53.
122. Lei YY, Ho SC, Kwok C, Cheng A, Cheung KL, Lee R, et al. Association of high adherence to vegetables and fruits dietary pattern with quality of life among Chinese women with early-stage breast cancer. Qual Life Res. 2022;31(5):1371-84.
123. Cheng Z, Anandavadivelan P, Nilsson M, Johar A, Lagergren P. Body Mass Index-Adjusted Weight Loss Grading System and Cancer-Related Fatigue in Survivors 1 Year After Esophageal Cancer Surgery. Ann Surg Oncol. 2022;29(7):4502-10.
124. Cheng Z, Johar A, Nilsson M, Lagergren P. Cancer-Related Fatigue After Esophageal Cancer Surgery: Impact of Postoperative Complications. Ann Surg Oncol. 2022;29(5):2842-51.
125. Singhal S, Walter LC, Smith AK, Loh KP, Cohen HJ, Zeng S, et al. Change in four measures of physical function among older adults during lung cancer treatment: A mixed methods cohort study. J Geriatr Oncol. 2023;14(2):101366.
126. Cella D, Sarda SP, Hsieh R, Fishman J, Hakimi Z, Hoffman K, et al. Changes in hemoglobin and clinical outcomes drive improvements in fatigue, quality of life, and physical function in patients with paroxysmal nocturnal hemoglobinuria: post hoc analyses from the phase III PEGASUS study. Annals of Hematology. 2022;101(9):1905-14.
127. Zhang J, Kong W, Hu P, Jonker D, Moore M, Ringash J, et al. Clustering on longitudinal quality-of-life measurements using growth mixture models for clinical prognosis: Implementation on CCTG/AGITG CO.20 trial. Cancer Medicine. 2023;12(5):6117-28.

128. Chang AY, Karwa R, Odhiambo H, Were P, Fletcher SL, Tonui EC, et al. Compression Therapy for HIV-Associated Kaposi Sarcoma Leg Lymphedema: Results of the Kenyan Improvised Compression for Kaposi Sarcoma Randomized Controlled Trial. JCO Glob Oncol. 2022;8:e2100329.
129. Meglio AD, Havas J, Soldato D, Presti D, Martin E, Pistilli B, et al. Development and Validation of a Predictive Model of Severe Fatigue After Breast Cancer Diagnosis: Toward a Personalized Framework in Survivorship Care. Journal of Clinical Oncology. 2022;40(10):1111-23.
130. Scott SI, Madsen AKØ, Rubek N, Charabi BW, Wessel I, Jensen CV, et al. Dysphagia and QoL 3 Years After Treatment of Oropharyngeal Cancer With TORS or Radiotherapy. The Laryngoscope. 2023;133(8):1893-8.
131. Oaknin A, Monk BJ, Vergote I, Cristina de Melo A, Kim Y-M, Lisyanskaya AS, et al. EMPOWER CERVICAL-1: Effects of cemiplimab versus chemotherapy on patient-reported quality of life, functioning and symptoms among women with recurrent cervical cancer. European Journal of Cancer. 2022;174:299-309.
132. Hanna L, Huggins CE, Furness K, Silvers MA, Savva J, Frawley H, et al. Effect of early and intensive nutrition care, delivered via telephone or mobile application, on quality of life in people with upper gastrointestinal cancer: study protocol of a randomised controlled trial. BMC Cancer. 2018;18(1):707.
133. Basch E, Schrag D, Henson S, Jansen J, Ginos B, Stover AM, et al. Effect of Electronic Symptom Monitoring on Patient-Reported Outcomes Among Patients With Metastatic Cancer: A Randomized Clinical Trial. JAMA. 2022;327(24):2413-22.
134. Hu Q, Xie B. Effect of Maitake D-fraction in advanced laryngeal and pharyngeal cancers during concurrent chemoradiotherapy: A randomized clinical trial. Acta Biochim Pol. 2022;69(3):625-32.
135. Tang L-L, Guo R, Zhang N, Deng B, Chen L, Cheng Z-B, et al. Effect of Radiotherapy Alone vs Radiotherapy With Concurrent Chemoradiotherapy on Survival Without Disease Relapse in Patients With Low-risk Nasopharyngeal Carcinoma: A Randomized Clinical Trial. JAMA. 2022;328(8):728-36.
136. Højer EG, Kreiberg M, Dehlendorff C, Jørgensen N, Juul A, Lauritsen J, et al. Effect of Testosterone Replacement Therapy on Quality of Life and Sexual Function in Testicular Cancer Survivors With Mild Leydig Cell Insufficiency: Results From a Randomized Double-blind Trial. Clinical Genitourinary Cancer. 2022;20(4):334-43.
137. Quintana JM, Anton-Ladislao A, Lázaro S, Gonzalez N, Bare M, Fernandez-de-Larrea N, et al. Effect of comorbidities on long-term outcomes of colorectal cancer patients. European Journal of Cancer Care. 2022;31(2):e13561.
138. Peng L, Yang Y, Chen M, Xu C, Chen Y, Liu R, et al. Effects of an online mindfulness-based intervention on Fear of Cancer Recurrence and quality of life among Chinese breast cancer survivors. Complementary Therapies in Clinical Practice. 2022;49:101686.
139. Weyhe D, Obonyo D, Uslar V, Tabriz N. Effects of intensive physiotherapy on Quality of Life (QoL) after pancreatic cancer resection: a randomized controlled trial. BMC Cancer. 2022;22(1):520.
140. Chen L, Tong F, Peng L, Huang Y, Yin P, Feng Y, et al. Efficacy and safety of recombinant human endostatin combined with whole-brain radiation therapy in patients with brain metastases from non-small cell lung cancer. Radiotherapy and Oncology. 2022;174:44-51.
141. Fischer J, Knop S, Danhof S, Einsele H, Keller D, Löffler C. The influence of baseline characteristics, treatment and depression on health-related quality of life in patients with multiple myeloma: a prospective observational study. BMC Cancer. 2022;22(1):1032.
142. Maio M, Amonkar MM, Norquist JM, Ascierto PA, Manzyuk L, Motola-Kuba D, et al. Health-related quality of life in patients treated with pembrolizumab for microsatellite instability-high/mismatch repair-deficient advanced solid tumours: Results from the KEYNOTE-158 study. Eur J Cancer. 2022;169:188-97.
143. Li T, Li B, Tan L, Lv B. Reminiscence Therapy as a Potential Method to Improve Psychological Health and Quality of Life in Elderly Hepatocellular Carcinoma Patients: A Randomized, Controlled Trial. Frontiers in Surgery. 2022;Volume 9 - 2022.
144. Palitzika D, Tilaveridis I, Lavdaniti M, Vahtsevanos K, Kosintzi A, Antoniades K. Quality of Life in Patients With Tongue Cancer After Surgical Treatment: A 12-Month Prospective Study. Cureus. 2022;14(2):e22511.
145. Adenis A, Kulkarni AS, Girotto GC, Fouchardiere Cdl, Senellart H, Laarhoven HWMv, et al. Impact of Pembrolizumab Versus Chemotherapy as Second-Line Therapy for Advanced Esophageal Cancer on Health-Related Quality of Life in KEYNOTE-181. Journal of Clinical Oncology. 2022;40(4):382-91.
146. Mey R, Casaña J, Díaz-Cambronero Ó, Suso-Martí L, Cuenca-Martínez F, Mazzinari G, et al. Physical and Quality of Life Changes in Elderly Patients after Laparoscopic Surgery for Colorectal Cancer-A Prospective Cohort Study. Int J Environ Res Public Health. 2022;19(22).
147. Wang YF, Wang TY, Liao TT, Lin MH, Huang TH, Hsieh MC, et al. Quality of life and symptom distress after cytoreductive surgery and hyperthermic intraperitoneal chemotherapy. World J Clin Cases. 2022;10(32):11775-88.

148. Ramim JE, Matheos de Lima BA, Bulzico DA, Pujatti PB, Bergmann A. Prospective Cohort Real-World Study on Neuroendocrine Tumor Patient's Quality of Life During Peptide Receptor Radionuclide Therapy With 177Lu-DOTATATE. Pancreas. 2022;51(7):784-9.
149. Mazieres J, Iadeluca L, Shaw AT, Solomon BJ, Bauer TM, de Marinis F, et al. Patient-reported outcomes from the randomized phase 3 CROWN study of first-line lorlatinib versus crizotinib in advanced ALK-positive non-small cell lung cancer. Lung Cancer. 2022;174:146-56.
150. Menon NS, Noronha V, Patil VM, Joshi A, Bhattacharjee A, Kalra D, et al. Quality of life in patients with locally advanced head and neck squamous cell carcinoma undergoing concurrent chemoradiation with once-a-week versus once-every-3-weeks cisplatin. Cancer Med. 2022;11(21):3939-48.
151. Schadewaldt V, Cherkil S, Panikar D, Drummond KJ. Quality of life after resection of a meningioma—A cross-cultural comparison of Indian and Australian patients. PLOS ONE. 2022;17(9):e0275184.
152. Lemij AA, van der Plas-Krijgsman WG, Bastiaannet E, Merkus JWS, van Dalen T, Vulink AJE, et al. Predicting postoperative complications and their impact on quality of life and functional status in older patients with breast cancer. British Journal of Surgery. 2022;109(7):595-602.
153. Schlichting M, Sanz H, Williams P, Ballarini N, Rippin G, Pawar V. Investigating the trends in patient-reported outcomes pre-treatment and implications to efficacy analyses: A post-hoc analysis of a cancer clinical trial. Contemporary Clinical Trials Communications. 2022;30:101021.
154. Agirrezabal I, Brennan VK, Colaone F, Shergill S, Pereira H, Chatellier G, et al. Transarterial Radioembolization Versus Atezolizumab-Bevacizumab in Unresectable Hepatocellular Carcinoma: A Matching-Adjusted Indirect Comparison of Time to Deterioration in Quality of Life. Adv Ther. 2022;39(5):2035-51.
155. Kristensen A, Grønberg BH, Fløtten Ø, Kaasa S, Solheim TS. Trajectory of health-related quality of life during the last year of life in patients with advanced non-small-cell lung cancer. Support Care Cancer. 2022;30(11):9351-8.
156. Lenz H-J, Van Cutsem E, Luisa Limon M, Wong KYM, Hendlisz A, Aglietta M, et al. First-Line Nivolumab Plus Low-Dose Ipilimumab for Microsatellite Instability-High/Mismatch Repair-Deficient Metastatic Colorectal Cancer: The Phase II CheckMate 142 Study. Journal of Clinical Oncology. 2021;40(2):161-70.
157. Tomanovic Vujadinovic S, Ilic N, Selakovic I, Nedeljkovic U, Krstic N, Mujovic N, et al. TENS Improves Cisplatin-Induced Neuropathy in Lung Cancer Patients. Medicina (Kaunas). 2022;58(10).
158. Aggarwal R, Alumkal JJ, Szmulewitz RZ, Higano CS, Bryce AH, Lopez-Gitlitz A, et al. Randomized, Open-Label Phase 2 Study of Apalutamide plus Androgen Deprivation Therapy versus Apalutamide Monotherapy versus Androgen Deprivation Monotherapy in Patients with Biochemically Recurrent Prostate Cancer. Prostate Cancer. 2022;2022(1):5454727.
159. Junker T, Duus L, Rasmussen BSB, Azawi N, Lund L, Nørgaard B, et al. Impact of Partial Nephrectomy and Percutaneous Cryoablation on Short-term Health-related Quality of Life—A Prospective Comparative Cohort Study. European Urology Open Science. 2022;45:99-107.
160. Tarkowska M, Głowacka-Mrotek I, Skonieczny B, Jankowski M, Nowikiewicz T, Jarzemski M, et al. Prospective Evaluation of the Quality of Life of Patients after Surgical Treatment of Rectal Cancer: A 12-Month Cohort Observation. J Clin Med. 2022;11(19).
161. Witjes JA, Galsky MD, Gschwend JE, Broughton E, Braverman J, Nasroulah F, et al. Health-related Quality of Life with Adjuvant Nivolumab After Radical Resection for High-risk Muscle-invasive Urothelial Carcinoma: Results from the Phase 3 CheckMate 274 Trial. Eur Urol Oncol. 2022;5(5):553-63.
162. Huang T, Su H, Zhang S, Huang Y. Reminiscence therapy-based care program serves as an optional nursing modality in alleviating anxiety and depression, improving quality of life in surgical prostate cancer patients. Int Urol Nephrol. 2022;54(10):2467-76.
163. Chase DM, Marín MR, Backes F, Han S, Graybill W, Mirza MR, et al. Impact of disease progression on health-related quality of life of advanced ovarian cancer patients – Pooled analysis from the PRIMA trial. Gynecologic Oncology. 2022;166(3):494-502.
164. Liu X, Yuan K, Ye X, Liu R. Proposing a novel care program: reminiscence therapy involved care for anxiety, depression, and quality of life in postoperative cervical cancer patients. Ir J Med Sci. 2022;191(5):2019-27.
165. Barrios CH, Saji S, Harbeck N, Zhang H, Jung KH, Patel S, et al. Patient-reported outcomes from a randomized trial of neoadjuvant atezolizumab-chemotherapy in early triple-negative breast cancer. npj Breast Cancer. 2022;8(1):108.
166. Chen Y, Xie Y, Zhang H, Li Z, Wu B, Li C, et al. Modified McKeown vs. traditional McKeown minimally invasive esophagectomy in improving short-term efficacy and the quality of life of esophageal cancers: a retrospective comparative cohort study. J Gastrointest Oncol. 2022;13(4):1579-88.

167. Bedke J, Rini BI, Plimack ER, Stus V, Gafanov R, Waddell T, et al. Health-related Quality of Life Analysis from KEYNOTE-426: Pembrolizumab plus Axitinib Versus Sunitinib for Advanced Renal Cell Carcinoma. European Urology. 2022;82(4):427-39.
168. Paramanandam VS, Dylke E, Clark GM, Daptardar AA, Kulkarni AM, Nair NS, et al. Prophylactic Use of Compression Sleeves Reduces the Incidence of Arm Swelling in Women at High Risk of Breast Cancer–Related Lymphedema: A Randomized Controlled Trial. Journal of Clinical Oncology. 2022;40(18):2004-12.
169. Villacampa G, Falato C, Paré L, Hernando C, Arumí M, Saura C, et al. Pre-operative ribociclib plus letrozole versus chemotherapy: Health-related quality of life outcomes from the SOLTI CORALLEEN trial. European Journal of Cancer. 2022;174:232-42.
170. Bulens PP, Smets L, Debucquoy A, Joye I, D'Hoore A, Wolthuis A, et al. Nonoperative versus operative approach according to the response to neoadjuvant chemoradiotherapy for rectal cancer: A prospective cohort study. Clinical and Translational Radiation Oncology. 2022;36:113-20.
171. Hamers PAH, Vink GR, Elferink MAG, Stellato RK, Dijksterhuis WPM, Punt CJA, et al. Quality of Life and Survival of Metastatic Colorectal Cancer Patients Treated With Trifluridine-Tipiracil (QUALITAS). Clin Colorectal Cancer. 2022;21(2):154-66.
172. O'Malley DM, Bariani GM, Cassier PA, Marabelle A, Hansen AR, De Jesus Acosta A, et al. Health-related quality of life with pembrolizumab monotherapy in patients with previously treated advanced microsatellite instability high/mismatch repair deficient endometrial cancer in the KEYNOTE-158 study. Gynecol Oncol. 2022;166(2):245-53.
173. Kim JH, Lee DE, Lee Y, Ha HI, Chang YJ, Chang SJ, et al. Quality of life outcomes from the randomized trial of hyperthermic intraperitoneal chemotherapy following cytoreductive surgery for primary ovarian cancer (KOV-HIPEC-01). J Gynecol Oncol. 2022;33(4):e54.
174. Walding A, Skaltsa K, Casamayor M, Rydén A. Time to deterioration of patient-reported outcomes in non-small cell lung cancer: exploring different definitions. Qual Life Res. 2022;31(8):2535-43.
175. Van Cutsem E, Kato K, Ajani J, Shen L, Xia T, Ding N, et al. Tislelizumab versus chemotherapy as second-line treatment of advanced or metastatic esophageal squamous cell carcinoma (RATIONALE 302): impact on health-related quality of life. ESMO Open. 2022;7(4):100517.
176. Kopetz S, Grothey A, Van Cutsem E, Yaeger R, Wasan H, Yoshino T, et al. Quality of life with encorafenib plus cetuximab with or without binimetinib treatment in patients with BRAF V600E-mutant metastatic colorectal cancer: patient-reported outcomes from BEACON CRC. ESMO Open. 2022;7(3):100477.
177. Araujo RO, Vieira FM, Victorino AP, Torres C, Martins I, Guaraldi S, et al. Quality of life in a randomized trial comparing two neoadjuvant regimens for locally advanced rectal cancer—INCAGI004. Supportive Care in Cancer. 2022;30(8):6557-72.
178. Ram R, Grisariu S, Shargian-Alon L, Amit O, Bar-On Y, Stepensky P, et al. Toxicity and efficacy of chimeric antigen receptor T-cell therapy in patients with diffuse large B-cell lymphoma above the age of 70 years compared to younger patients - a matched control multicenter cohort study. Haematologica. 2022;107(5):1111-8.
179. Presti D, Havas J, Soldato D, Lapidari P, Martin E, Pistilli B, et al. Factors associated with enrolment in clinical trials among women with early-stage breast cancer. ESMO Open. 2022;7(3):100513.
180. van der Doelen MJ, Oving IM, Wyndaele DNJ, van Basten J-P, Terheggen F, van de Luijtgaarden ACM, et al. Health-related quality of life, psychological distress, and fatigue in metastatic castration-resistant prostate cancer patients treated with radium-223 therapy. Prostate Cancer and Prostatic Diseases. 2023;26(1):142-50.
181. Verweij ME, Hoendervangers S, Couwenberg AM, Burbach JPM, Berbee M, Buijsen J, et al. Impact of Dose-Escalated Chemoradiation on Quality of Life in Patients With Locally Advanced Rectal Cancer: 2-Year Follow-Up of the Randomized RECTAL-BOOST Trial. International Journal of Radiation Oncology*Biology*Physics. 2022;112(3):694-703.
182. Abel E, Silander E, Nordström F, Olsson C, Brodin NP, Nyman J, et al. Fatigue in Patients With Head and Neck Cancer Treated With Radiation Therapy: A Prospective Study of Patient-Reported Outcomes and Their Association With Radiation Dose to the Cerebellum. Advances in Radiation Oncology. 2022;7(5):100960.
183. Riccetti N, Felberbaum R, Flock F, Kühn T, Leinert E, Schwentner L, et al. Financial difficulties in breast cancer survivors with and without migration background in Germany-results from the prospective multicentre cohort study BRENDA II. Support Care Cancer. 2022;30(8):6677-88.
184. Emine KE, Gulbeyaz C. The effect of salt-water bath in the management of treatment-related peripheral neuropathy in cancer patients receiving taxane and platinum-based treatment. EXPLORE. 2022;18(3):347-56.

185. Ahn GS, Hwang K, Kim TM, Park CK, Chang JH, Jung TY, et al. Influence of Concurrent and Adjuvant Temozolomide on Health-Related Quality of Life of Patients with Grade III Gliomas: A Secondary Analysis of a Randomized Clinical Trial (KNOG-1101 Study). Cancer Res Treat. 2022;54(2):396-405.
186. Smedman TM, Guren TK, Tveit KM, Thomsen M, Andersen MH, Line P-D, et al. Health-Related Quality of Life in Colorectal Cancer Patients Treated With Liver Transplantation Compared to Chemotherapy. Transplant International. 2022;Volume 35 - 2022.
187. Dal Lago L, Uwimana AL, Coens C, Vuylsteke P, Curigliano G, Brouwers B, et al. Health-related quality of life in older patients with HER2+ metastatic breast cancer: Comparing pertuzumab plus trastuzumab with or without metronomic chemotherapy in a randomised open-label phase II clinical trial. Journal of Geriatric Oncology. 2022;13(5):582-93.
188. Langlais CS, Chen Y-H, Van Blarigan EL, Kenfield SA, Kessler ER, Daniel K, et al. Quality of Life of Prostate Cancer Survivors Participating in a Remotely Delivered Web-Based Behavioral Intervention Pilot Randomized Trial. Integrative Cancer Therapies. 2022;21:15347354211063500.
189. van Amelsfoort RM, Walraven I, Kieffer J, Jansen EPM, Cats A, van Grieken NCT, et al. Quality of Life Is Associated With Survival in Patients With Gastric Cancer: Results From the Randomized CRITICS Trial. J Natl Compr Canc Netw. 2022;20(3):261-7.
190. Lu S, Yu Y, Barnes G, Qiu X, Bao Y, Tang B. Examining the Impact of Tislelizumab Added to Chemotherapy on Health-Related Quality-of-Life Outcomes in Previously Untreated Patients With Nonsquamous Non–Small Cell Lung Cancer. The Cancer Journal. 2022;28(2):96-104.
191. Pratz KW, Panayiotidis P, Recher C, Wei X, Jonas BA, Montesinos P, et al. Venetoclax combinations delay the time to deterioration of HRQoL in unfit patients with acute myeloid leukemia. Blood Cancer Journal. 2022;12(4):71.
192. Ax AK, Johansson B, Lyth J, Nordin K, Börjeson S. Short- and long-term effect of high versus low-to-moderate intensity exercise to optimise health-related quality of life after oncological treatment-results from the Phys-Can project. Support Care Cancer. 2022;30(7):5949-63.
193. Bertaut A, Touchefeu Y, Blanc J, Bouché O, François E, Conroy T, et al. Health-Related Quality of Life Analysis in Metastatic Colorectal Cancer Patients Treated by Second-Line Chemotherapy, Associated With Either Cetuximab or Bevacizumab: The PRODIGE 18 Randomized Phase II Study. Clinical Colorectal Cancer. 2022;21(2):e49-e61.
194. Micaux C, Wiklander M, Eriksson LE, Wettergren L, Lampic C. Efficacy of a Web-Based Psychoeducational Intervention for Young Adults With Fertility-Related Distress Following Cancer (Fex-Can): Randomized Controlled Trial. JMIR Cancer. 2022;8(1):e33239.
195. Motzer R, Porta C, Alekseev B, Rha SY, Choueiri TK, Mendez-Vidal MJ, et al. Health-related quality-of-life outcomes in patients with advanced renal cell carcinoma treated with lenvatinib plus pembrolizumab or everolimus versus sunitinib (CLEAR): a randomised, phase 3 study. The Lancet Oncology. 2022;23(6):768-80.
196. Liu ZP, Song QY, Chu CL, Liu SH, Ren YX, Chen J, et al. Efficacy of Kanglaite against radiotherapy-induced mucositis in head and neck cancer, a phase II trial. Cancer/Radiothérapie. 2022;26(4):585-93.
197. Rush HL, Murphy L, Morgans AK, Clarke NW, Cook AD, Attard G, et al. Quality of Life in Men With Prostate Cancer Randomly Allocated to Receive Docetaxel or Abiraterone in the STAMPEDE Trial. J Clin Oncol. 2022;40(8):825-36.
198. Sundar S, Cummins C, Kumar S, Long J, Arora V, Balega J, et al. Quality of life from cytoreductive surgery in advanced ovarian cancer: Investigating the association between disease burden and surgical complexity in the international, prospective, SOCQER-2 cohort study. Bjog. 2022;129(7):1122-32.
199. Stockler MR, Martin AJ, Davis ID, Dhillon HM, Begbie SD, Chi KN, et al. Health-Related Quality of Life in Metastatic, Hormone-Sensitive Prostate Cancer: ENZAMET (ANZUP 1304), an International, Randomized Phase III Trial Led by ANZUP. Journal of Clinical Oncology. 2021;40(8):837-46.
200. Wen N, Ouyang C, Hu X, Hou L, He L, Liu C, et al. What Is the Optimal Strategy for Drain Removal After Mastectomy and Axillary Surgery in Breast Cancer Patients? A Multicenter, Three-Arm Randomized Clinical Trial. Journal of Surgical Research. 2022;277:148-56.
201. Li J, Cheng Y, Bai C, Xu J, Shen L, Li J, et al. Health-related quality of life in patients with advanced well-differentiated pancreatic and extrapancreatic neuroendocrine tumors treated with surufatinib versus placebo: Results from two randomized, double-blind, phase III trials (SANET-p and SANET-ep). European Journal of Cancer. 2022;169:1-9.
202. van der Wielen N, Daams F, Rosati R, Parise P, Weitz J, Reissfelder C, et al. Health related quality of life following open versus minimally invasive total gastrectomy for cancer: Results from a randomized clinical trial. European Journal of Surgical Oncology. 2022;48(3):553-60.

203. Takahashi M, Hwang M, Misiukiewicz K, Gupta V, Miles BA, Bakst R, et al. Quality of Life Analysis of HPV-Positive Oropharyngeal Cancer Patients in a Randomized Trial of Reduced-Dose Versus Standard Chemoradiotherapy: 5-Year Follow-Up. Frontiers in Oncology. 2022;Volume 12 - 2022.
204. Yu J, Wang Z, Li Z, Liu Y, Fan Y, Di J, et al. Health-Related Quality of Life in Patients With Locally Advanced Gastric Cancer Undergoing Perioperative or Postoperative Adjuvant S-1 Plus Oxaliplatin With D2 Gastrectomy: A Propensity Score-Matched Cohort Study. Frontiers in Oncology. 2022;Volume 12 - 2022.
205. McGregor B, O'Donnell PH, Balar A, Petrylak D, Rosenberg J, Yu EY, et al. Health-related Quality of Life of Patients with Locally Advanced or Metastatic Urothelial Cancer Treated with Enfortumab Vedotin after Platinum and PD-1/PD-L1 Inhibitor Therapy: Results from Cohort 1 of the Phase 2 EV-201 Clinical Trial. European Urology. 2022;81(5):515-22.
206. Wortman BG, Post CCB, Powell ME, Khaw P, Fyles A, D'Amico R, et al. Radiation Therapy Techniques and Treatment-Related Toxicity in the PORTEC-3 Trial: Comparison of 3-Dimensional Conformal Radiation Therapy Versus Intensity-Modulated Radiation Therapy. International Journal of Radiation Oncology*Biology*Physics. 2022;112(2):390-9.
207. Tang L-L, Huang C-L, Zhang N, Jiang W, Wu Y-S, Huang SH, et al. Elective upper-neck versus whole-neck irradiation of the uninvolved neck in patients with nasopharyngeal carcinoma: an open-label, non-inferiority, multicentre, randomised phase 3 trial. The Lancet Oncology. 2022;23(4):479-90.
208. Zhang Q, Wang Y, Yang S, Wu Q, Qiang W. What is the appropriate skin cleaning method for nasopharyngeal cancer radiotherapy patients? A randomized controlled trial. Supportive Care in Cancer. 2022;30(5):3875-83.
209. Rajamanickam S, Chaukar D, Siddiq S, Basu S, D’Cruz A. Quality of life comparison in thyroxine hormone withdrawal versus triiodothyronine supplementation prior to radioiodine ablation in differentiated thyroid carcinoma: a prospective cohort study in the Indian population. European Archives of Oto-Rhino-Laryngology. 2022;279(4):2011-8.
210. Kutz LM, Abel J, Schweizer D, Tribius S, Krüll A, Petersen C, et al. Quality of life, HPV-status and phase angle predict survival in head and neck cancer patients under (chemo)radiotherapy undergoing nutritional intervention: Results from the prospective randomized HEADNUT-trial. Radiotherapy and Oncology. 2022;166:145-53.
211. O'Gara G, Murray L, Georgopoulou S, Anstiss T, Macquarrie A, Wheatstone P, et al. SafeSpace: what is the feasibility and acceptability of a codesigned virtual reality intervention, incorporating compassionate mind training, to support people undergoing cancer treatment in a clinical setting? BMJ Open. 2022;12(2):e047626.
212. Wang J, Yu X, Barnes G, Leaw S, Bao Y, Tang B. The effects of tislelizumab plus chemotherapy as first-line treatment on health-related quality of life of patients with advanced squamous non-small cell lung cancer: Results from a phase 3 randomized clinical trial. Cancer Treatment and Research Communications. 2022;30:100501.
213. Williams AM, Kathawate RG, Zhao L, Grenda TR, Bergquist CS, Brescia AA, et al. Similar Quality of Life After Conventional and Robotic Transhiatal Esophagectomy. The Annals of Thoracic Surgery. 2022;113(2):399-405.
214. O'Hara J, Whitmarsh A, Pring M, Thomas S, Ness A. Quality of life following treatment for T1a glottic cancer with surgery or radiotherapy: outcomes from the Head and Neck 5000 cohort. Clin Otolaryngol. 2022;47(1):67-74.
215. Lei Y-Y, Ho SC, Lau TKH, Kwok C, Cheng A, Cheung KL, et al. Longitudinal change of quality of life in the first five years of survival among disease-free Chinese breast cancer survivors. Quality of Life Research. 2021;30(6):1583-94.
216. Ichimura H, Kobayashi K, Gosho M, Nakaoka K, Yanagihara T, Ueda S, et al. Preoperative predictors of restoration in quality of life after surgery for lung cancer. Thoracic Cancer. 2021;12(6):835-44.
217. Morgan JL, Shrestha A, Reed MWR, Herbert E, Bradburn M, Walters SJ, et al. Bridging the age gap in breast cancer: impact of omission of breast cancer surgery in older women with oestrogen receptor-positive early breast cancer on quality-of-life outcomes. British Journal of Surgery. 2021;108(3):315-25.
218. Zhao H, Yao W, Min X, Gu K, Yu G, Zhang Z, et al. Apatinib Plus Gefitinib as First-Line Treatment in Advanced EGFR-Mutant NSCLC: The Phase III ACTIVE Study (CTONG1706). Journal of Thoracic Oncology. 2021;16(9):1533-46.
219. Zhang H, Jiang M, Gao L, Lin Z. The clinical efficacy of external application of mirabilite and rhubarb combined with intrathoracic chemotherapy in treating malignant pleural effusion: A prospective, randomized, controlled clinical trial. Medicine (Baltimore). 2021;100(7):e24758.

220. Jaeckle KA, Ballman KV, van den Bent M, Giannini C, Galanis E, Brown PD, et al. CODEL: phase III study of RT, RT + TMZ, or TMZ for newly diagnosed 1p/19q codeleted oligodendroglioma. Analysis from the initial study design. Neuro Oncol. 2021;23(3):457-67.
221. Pattamatta M, C. FLF, P. D-ZAC, P. NGA, A. ESMA, A. KE, et al. Effect of direct oral feeding following minimally invasive esophagectomy on costs and quality of life. Journal of Medical Economics. 2021;24(1):54-60.
222. Asrar MM, P. LD, Dipika B, Shankar P, Alka K, Gaurav P, et al. Health-related quality of life in transplant eligible multiple myeloma patients with or without early ASCT in the real-world setting. Leukemia & Lymphoma. 2021;62(13):3271-7.
223. Korrel M, Roelofs A, van Hilst J, Busch OR, Daams F, Festen S, et al. Long-Term Quality of Life after Minimally Invasive vs Open Distal Pancreatectomy in the LEOPARD Randomized Trial. Journal of the American College of Surgeons. 2021;233(6):730-9.e9.
224. Jansen MR, Vrielink OM, Faut M, Deckers EA, Been LB, van Leeuwen BL. One-Year Morbidity Following Videoscopic Inguinal Lymphadenectomy for Stage III Melanoma. Cancers (Basel). 2021;13(6).
225. Adamson D, Byrne A, Porter C, Blazeby J, Griffiths G, Nelson A, et al. Palliative radiotherapy after oesophageal cancer stenting (ROCS): a multicentre, open-label, phase 3 randomised controlled trial. The Lancet Gastroenterology & Hepatology. 2021;6(4):292-303.
226. Van den Bosch L, van der Laan HP, van der Schaaf A, Oosting SF, Halmos GB, Witjes MJH, et al. Patient-Reported Toxicity and Quality-of-Life Profiles in Patients With Head and Neck Cancer Treated With Definitive Radiation Therapy or Chemoradiation. Int J Radiat Oncol Biol Phys. 2021;111(2):456-67.
227. Marinello FG, Jiménez LM, Talavera E, Fraccalvieri D, Alberti P, Ostiz F, et al. Percutaneous tibial nerve stimulation in patients with severe low anterior resection syndrome: randomized clinical trial. British Journal of Surgery. 2021;108(4):380-7.
228. Vittrup AS, Tanderup K, Bentzen SM, Jensen NBK, Spampinato S, Fokdal LU, et al. Persistence of Late Substantial Patient-Reported Symptoms (LAPERS) After Radiochemotherapy Including Image Guided Adaptive Brachytherapy for Locally Advanced Cervical Cancer: A Report From the EMBRACE Study. International Journal of Radiation Oncology*Biology*Physics. 2021;109(1):161-73.
229. Gogas H, Dummer R, Ascierto PA, Arance A, Mandalà M, Liszkay G, et al. Quality of life in patients with BRAF-mutant melanoma receiving the combination encorafenib plus binimetinib: Results from a multicentre, open-label, randomised, phase III study (COLUMBUS). European Journal of Cancer. 2021;152:116-28.
230. Wortman B, Post C, Powell M, Khaw P, Fyles A, D'Amico R, et al. Radiotherapy Techniques and Treatment-Related Toxicity in the PORTEC-3 Trial: Comparison of Three-Dimensional Conformal Radiotherapy versus Intensity-Modulated Radiotherapy. International Journal of Radiation Oncology*Biology*Physics. 2021;112.
231. Le Cesne A, Blay JY, Cupissol D, Italiano A, Delcambre C, Penel N, et al. A randomized phase III trial comparing trabectedin to best supportive care in patients with pre-treated soft tissue sarcoma: T-SAR, a French Sarcoma Group trial. Annals of Oncology. 2021;32(8):1034-44.
232. Wu O, McCartney E, Heggie R, Germeni E, Paul J, Soulis E, et al. Venous access devices for the delivery of long-term chemotherapy: the CAVA three-arm RCT. Health Technol Assess. 2021;25(47):1-126.
233. Gregersen H, Peceliunas V, Remes K, Schjesvold F, Abildgaard N, Nahi H, et al. Carfilzomib and dexamethasone maintenance following salvage ASCT in multiple myeloma: A randomised phase 2 trial by the Nordic Myeloma Study Group. Eur J Haematol. 2022;108(1):34-44.
234. Gogishvili M, Melkadze T, Makharadze T, Giorgadze D, Dvorkin M, Penkov K, et al. Cemiplimab plus chemotherapy versus chemotherapy alone in non-small cell lung cancer: a randomized, controlled, double-blind phase 3 trial. Nat Med. 2022;28(11):2374-80.
235. Hosoi T, Abe T, Higaki E, Fujieda H, Nagao T, Ito S, et al. Circular Stapled Technique Versus Modified Collard Technique for Cervical Esophagogastric Anastomosis After Esophagectomy: A Randomized Controlled Trial. Annals of Surgery. 2022;276(1).
236. Yang L, Yan C, Wang J. Effect of multi-disciplinary team care program on quality of life, anxiety, and depression in hepatocellular carcinoma patients after surgery: A randomized, controlled study. Frontiers in Surgery. 2023;Volume 9 - 2022.
237. Sanchorawala V, Palladini G, Minnema MC, Jaccard A, Lee HC, Gibbs S, et al. Health-related quality of life in patients with light chain amyloidosis treated with bortezomib, cyclophosphamide, and dexamethasone ± daratumumab: Results from the ANDROMEDA study. American Journal of Hematology. 2022;97(6):719-30.
238. Oliva EN, Platzbecker U, Garcia-Manero G, Mufti GJ, Santini V, Sekeres MA, et al. Health-Related Quality of Life Outcomes in Patients with Myelodysplastic Syndromes with Ring Sideroblasts Treated with Luspatercept in the MEDALIST Phase 3 Trial. J Clin Med. 2021;11(1).

239. Forner LE, Dieleman FJ, Shaw RJ, Kanatas A, Butterworth CJ, Kjeller G, et al. Hyperbaric oxygen treatment of mandibular osteoradionecrosis: Combined data from the two randomized clinical trials DAHANCA-21 and NWHHT2009-1. Radiotherapy and Oncology. 2022;166:137-44.
240. Lim E, Harris RA, McKeon HE, Batchelor TJ, Dunning J, Shackcloth M, et al. Impact of video-assisted thoracoscopic lobectomy versus open lobectomy for lung cancer on recovery assessed using self-reported physical function: VIOLET RCT. Health Technol Assess. 2022;26(48):1-162.
241. Garcia Campelo MR, Zhou C, Ramalingam SS, Lin HM, Kim TM, Riely GJ, et al. Mobocertinib (TAK-788) in EGFR Exon 20 Insertion+ Metastatic NSCLC: Patient-Reported Outcomes from EXCLAIM Extension Cohort. J Clin Med. 2022;12(1).
242. Mercieca-Bebber R, Barnes EH, Wilson K, Samoon Z, Walpole E, Mai T, et al. Patient-reported outcome (PRO) results from the AGITG DOCTOR trial: a randomised phase 2 trial of tailored neoadjuvant therapy for resectable oesophageal adenocarcinoma. BMC Cancer. 2022;22(1):276.
243. Schöffski P, George S, Heinrich MC, Zalcberg JR, Bauer S, Gelderblom H, et al. Patient-reported outcomes in individuals with advanced gastrointestinal stromal tumor treated with ripretinib in the fourth-line setting: analysis from the phase 3 INVICTUS trial. BMC Cancer. 2022;22(1):1302.
244. Elsawy M, Chavez JC, Avivi I, Larouche J-F, Wannesson L, Cwynarski K, et al. Patient-reported outcomes in ZUMA-7, a phase 3 study of axicabtagene ciloleucel in second-line large B-cell lymphoma. Blood. 2022;140(21):2248-60.
245. Rammant E, Van Hecke A, Van Cauwenberg J, Decaestecker K, Poppe L, Russel B, et al. Physical Activity and Health-related Quality of Life from Diagnosis to One Year After Radical Cystectomy in Patients with Bladder Cancer: A Longitudinal Cohort Study. Bladder Cancer. 2022;8(4):395-404.
246. Kang SH, Yoo M, Hwang D, Lee E, Lee S, Park YS, et al. Postoperative pain and quality of life after single-incision distal gastrectomy versus multiport laparoscopic distal gastrectomy for early gastric cancer - a randomized controlled trial. Surg Endosc. 2023;37(3):2095-103.
247. Pappou EP, Temple LK, Patil S, Smith JJ, Wei IH, Nash GM, et al. Quality of life and function after rectal cancer surgery with and without sphincter preservation. Front Oncol. 2022;12:944843.
248. Lavdaniti M, Tilaveridis I, Palitzika D, Kyrgidis A, Triaridis S, Vachtsevanos K, et al. Quality of Life in Oral Cancer Patients in Greek Clinical Practice: A Cohort Study. J Clin Med. 2022;11(23).
249. Zinzani PL, Ramchandren R, Santoro A, Paszkiewicz-Kozik E, Gasiorowski R, Johnson NA, et al. Quality-of-life analysis of pembrolizumab vs brentuximab vedotin for relapsed/refractory classical Hodgkin lymphoma. Blood Adv. 2022;6(2):590-9.
250. Gilbert A, Homer V, Brock K, Korsgen S, Geh I, Hill J, et al. Quality-of-life outcomes in older patients with early-stage rectal cancer receiving organ-preserving treatment with hypofractionated short-course radiotherapy followed by transanal endoscopic microsurgery (TREC): non-randomised registry of patients unsuitable for total mesorectal excision. Lancet Healthy Longev. 2022;3(12):e825-e38.
251. Shiroiwa T, Hagiwara Y, Taira N, Kawahara T, Konomura K, Iwamoto T, et al. Randomized Controlled Trial of Paper-Based at a Hospital versus Continual Electronic Patient-Reported Outcomes at Home for Metastatic Cancer Patients: Does Electronic Measurement at Home Detect Patients' Health Status in Greater Detail? Medical Decision Making. 2021;42(1):60-7.
252. Chen L, Yang X, Ren X, Lin Y. Reminiscence therapy care program as a potential nursing intervention to relieve anxiety, depression, and quality of life in older papillary thyroid carcinoma patients: A randomized, controlled study. Front Psychol. 2022;13:1064439.
253. Accogli MA, Denti M, Costi S, Fugazzaro S. Therapeutic education and physical activity are feasible and safe in hematologic cancer patients referred to chemotherapy: results of a randomized controlled trial. Support Care Cancer. 2022;31(1):61.
254. Kawahara T, Taira N, Shiroiwa T, Hagiwara Y, Fukuda T, Uemura Y, et al. Minimal important differences of EORTC QLQ-C30 for metastatic breast cancer patients: Results from a randomized clinical trial. Qual Life Res. 2022;31(6):1829-36.
255. Koller M, Musoro JZ, Tomaszewski K, Coens C, King MT, Sprangers MAG, et al. Minimally important differences of EORTC QLQ-C30 scales in patients with lung cancer or malignant pleural mesothelioma – Interpretation guidance derived from two randomized EORTC trials. Lung Cancer. 2022;167:65-72.
256. Chatterjee S, Maulik S, Prasath S, Arun B, Das A, Chakrabarty S, et al. Xerostomia quality of life and resource requirements following parotid sparing adaptive radiotherapy in head and neck cancers: Results of a prospective cohort study (Study ID CTRI/2017/11/010683). Radiotherapy and Oncology. 2022;168:250-5.
257. Hosni A, Ringash J, Han K, Liu ZA, Brierley JD, Wong RKS, et al. Impact of Definitive Chemoradiation on Quality-of-Life Changes for Patients With Anal Cancer: Long-term Results of a Prospective Study. Dis Colon Rectum. 2022;65(5):642-53.

258. Pereira H, Bouattour M, Dioguardi Burgio M, Assenat E, Grégory J, Bronowicki JP, et al. Health-related quality of life in locally advanced hepatocellular carcinoma treated by either radioembolisation or sorafenib (SARAH trial). Eur J Cancer. 2021;154:46-56.
259. Lehman M, Bernard A, See A, King M, Michael M. A Randomized Phase 3 Trial of Palliative Radiation Therapy Versus Concurrent Chemotherapy and Palliative Radiation Therapy in Patients With Good Performance Status, Locally Advanced, or Metastatic Non-Small Cell Lung Cancer With Symptoms due to Intrathoracic Disease Who are Not Suitable for Radical Chemo-radiation Therapy: Results of the Trans-Tasman Radiation Oncology Group 11.03 Trial. Pract Radiat Oncol. 2021;11(4):252-63.
260. Author details unknown. Effect of whole-course nursing management on postoperative recovery and complications of enterostomy patients. Acta Medica Mediterranea. 2022.
261. Kawashima Y, Fukuhara T, Saito H, Furuya N, Watanabe K, Sugawara S, et al. Bevacizumab plus erlotinib versus erlotinib alone in Japanese patients with advanced, metastatic, EGFR-mutant non-small-cell lung cancer (NEJ026): overall survival analysis of an open-label, randomised, multicentre, phase 3 trial. Lancet Respir Med. 2022;10(1):72-82.
262. Mazza GL, Mead-Harvey C, Mascarenhas J, Yacoub A, Kosiorek HE, Hoffman R, et al. Symptom burden and quality of life in patients with high-risk essential thrombocythaemia and polycythaemia vera receiving hydroxyurea or pegylated interferon alfa-2a: a post-hoc analysis of the MPN-RC 111 and 112 trials. Lancet Haematol. 2022;9(1):e38-e48.
263. Vogel A, Qin S, Kudo M, Su Y, Hudgens S, Yamashita T, et al. Lenvatinib versus sorafenib for first-line treatment of unresectable hepatocellular carcinoma: patient-reported outcomes from a randomised, open-label, non-inferiority, phase 3 trial. Lancet Gastroenterol Hepatol. 2021;6(8):649-58.
264. Lattimore CM, Meneveau MO, Petroni GR, Varhegyi NE, Squeo GC, Showalter TN, et al. Effects of a novel form of intraoperative radiation therapy on quality of life among patients with early-stage breast cancer. Brachytherapy. 2022;21(3):325-33.
265. Ben-Arye E, Gamus D, Samuels N, Schiff E, Hausner D, Gressel O, et al. Acupuncture and integrative oncology for taxane-induced peripheral neuropathy: a randomized multicentered study. Int J Gynecol Cancer. 2023;33(5):792-801.
266. Laigle-Donadey F, Metellus P, Guyotat J, Menei P, Proust F, Dufour H, et al. Surgery for glioblastomas in the elderly: an Association des Neuro-oncologues d'Expression Française (ANOCEF) trial. J Neurosurg. 2023;138(5):1199-205.
267. Seven M, Paşalak Ş, Bagcivan G, Ozkasap O, Selçukbirick F. A Mobile Application for Symptom Management in Patients With Breast Cancer. Oncol Nurs Forum. 2022;49(5):409-20.
268. Giani C, Valerio L, Bongiovanni A, Durante C, Grani G, Ibrahim T, et al. Safety and Quality-of-Life Data from an Italian Expanded Access Program of Lenvatinib for Treatment of Thyroid Cancer. Thyroid. 2021;31(2):224-32.
269. Heino P, Mylläri P, Jahkola T, Luoma ML, Räsänen P, Roine RP. Surgery, Limb Edema and Health-related Quality of Life: A Prospective Follow-up Study on Patients With Cutaneous Malignant Melanoma. Anticancer Res. 2022;42(11):5507-19.
270. Alimena S, Philp L, Orav EJ, Sullivan MW, Del Carmen M, Goodman A, et al. Patient-reported outcomes and chemotherapy-related cognitive impairment in gynecologic malignancy. Int J Gynecol Cancer. 2022;32(6):781-7.
271. Vähäaho N, Hakamies-Blomqvist L, Blomqvist C, Kellokumpu-Lehtinen PL, Huovinen R, Saarto T, et al. Sense of Coherence as Predictor of Quality of Life in Early Breast Cancer Patients. Anticancer Res. 2021;41(10):5045-52.
